# Metal free C–O bond cleavage: a new strategy for the synthesis of substituted oxazoles†

**DOI:** 10.1039/d4ra05122j

**Published:** 2024-09-04

**Authors:** Shengwang Li, Guiqin Liu, Zheyan Zhang, Ruiling Chen, Haiying Tian, Huifeng Wang, Xiuling Chen

**Affiliations:** a Hubei Key Laboratory of Radiation Chemistry and Functional Materials, Hubei University of Science and Technology Xianning 437100 China cxl828800@163.com (+)86-715-8338007; b School of Pharmacy, Changzhi Medical College Changzhi 046000 China

## Abstract

A strategy for the efficient metal-free C–O bond cleavage of ester using amines for the synthesis of substituted oxazoles was developed for the first time. The synthesis proceeded smoothly under metal-free conditions, combining C–O bond cleavage as well as C–N and C–O bond formation in one pot to yield desired products in moderate to excellent yields, and accommodated a wide range of functional groups and substrates.

## Abstract 2

A strategy for C–O bond cleavage of ester for the synthesis of substituted oxazoles was developed. The synthesis proceeded smoothly under metal-free conditions, combining C–O bond cleavage, C–N and C–O bond formation in one pot.

## Graphical abstract

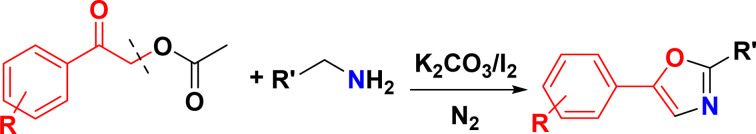

## Introduction

The cleavage of strong C–O bonds and the transformation of biomass into valuable compounds have attracted the attention of many researchers.^1,2^ Currently, C–O bond cleavage is mainly concentrated on phenols, ethers, and alcohols.^3–5^ However, there are relatively few reports on C–O bond cleavage of esters under metal-free conditions.^6^ Therefore, the development of a new strategy for C–O bond cleavage of esters is a primary research endeavor sought by many in modern chemical science. Metal-free-catalyzed reactions are among the most magnetic synthetic methods.^7^ With our continued interest in C–O bond cleavage,^8^ we aim to develop new methods for C–O cleavage and transformation under metal-free conditions.

Heterocyclic compounds play a crucial role in drug discovery and medicinal chemistry owing to their unique structural properties and biological activities.^9–12^ Five-membered heterocyclic compounds containing oxygen and nitrogen atoms, such as substituted oxazoles, are prominent heterocyclic structures. Substituted oxazole is a prominent five-membered heterocyclic structure that widely exists in plenty of natural products, drugs, and biological compounds and exhibits activity against diabetes, Gram-positive and Gram-negative bacterial infections, breast cancer, and pancreatic cancer. For example, oxaprozin, a substituted oxazole, is widely used to treat rheumatoid arthritis, osteoarthritis, ankylosing spondylitis, cervical spondylosis, and periarthritis of the shoulder.^9–12^ The synthesis of substituted oxazole derivatives has attracted increasing attention owing to the unique properties of the oxazole moiety. Recently, a variety of highly efficient methods have been reported for the synthesis of substituted oxazoles, such as cyclization of benzylamines with 1,2-dicarbonyls, 1,3-dicarbonyls, α-bromo ketones, aldehydes, alkenes, chalcones, ketones,^13–19^ and other moieties.^20^ Although multitudinous efficient methods have been developed to prepare substituted oxazoles, the substrate scope of amines is mainly limited to benzylamine. Recently, Li's group developed a novel method for the preparation of substituted oxazoles *via* CO_2_/photoredox-cocatalyzed tandem oxidative cyclization of α-bromo ketones and amines. Alkyl-substituted amines were also tolerated in this reaction. However, since CO_2_ gas and eosin Y were used in the reaction system, the photocatalyst suffered from poor stability and was easily destroyed by light and oxidants, thus remaining in drug molecules. Additionally, the reaction rate was slow and required a longer exposure to light. Hence, the exploration of more efficient methods for the preparation of substituted oxazoles is in continuous demand.

Herein, we developed a novel method for substituted oxazole synthesis through cyclization of substituted 2-oxo-2-phenylethyl acetate and amines *via* highly chemoselective C–O bond cleavage as well as C–N and C–O bond formation in one pot (eqn (1)). Compared with the existing methods, we expanded the substrate scope and developed a novel ester and alkyl amine as a substrate for the synthesis of substituted oxazole with moderate to good yields under metal-free conditions. This protocol offers an environmentally benign process and accommodates a broad substrate scope. Benzylamines bear electron-donating or electron-withdrawing groups and aliphatic amines to give the corresponding substituted oxazole compounds in good to excellent yields.

## Results and discussion

2-Oxo-2-phenylethyl acetate (1a) and benzylamine (2a) served as model reactants for an initial optimization study. To optimize reaction conditions, our initial efforts focused on screening solvents in the presence of equivalent I_2_/K_2_CO_3_ systems (the results of the other bases are in Table S1†), and the results are summarized in Table 1. Use of various anhydrous solvents showed that the reaction does not occur in non-polar solvents such as 1,4-dioxane, toluene, chlorobenzene trace, 1,2-dichloroethane (Table 1, entries 1–4), and the reaction proceeds smoothly in polar solvents such as acetonitrile *N*,*N*-dimethyl formamide, dimethylsulfoxide, ethyl acetate (Table 1, entries 5–8). This implies that a relatively polar solvent is necessary to dissolve K_2_CO_3_ or a nucleophilic substitution reaction may be involved. The content of I_2_ and K_2_CO_3_ had a great influence on the reaction: in the absence of K_2_CO_3_ (0.4 mmol) and with 0.1 mmol of I_2_, compound 3a was not detected. Likewise, in the absence of I_2_ (0.4 mmol) and with 0.1 mmol of K_2_CO_3_, compound 3a was not detected (Table 1, entry 10). The best result was obtained by carrying out the reaction with 0.4 mmol K_2_CO_3_ and I_2_ (Table 1, entry 9). Moreover, 3a was not detected when K_2_CO_3_ and I_2_ were absent from the reaction system (Table 1, entries 11 and 12). Thus, both the presence and the amounts of I_2_ and K_2_CO_3_ are essential for the reaction. The present reaction also depends on temperature: product 3a was not detected when the reaction was carried out at room temperature (Table 1, entry 13), and only 35% yield of 3a was obtained at 50 °C (Table 1, entry 14). Upon raising the temperature to 110 °C, 3a with only 78% yield was obtained. This is probably because the high temperatures led to the side reactions of benzylamine (Table 1, entry 15). It was noteworthy that other bases such as Cs_2_CO_3_, KOH, and DBU were all ineffective (Table 1, entries 16–18). Thus, the optimized conditions for the synthesis of 3a can be defined as follows: substrate = 2-oxo-2-phenylethyl acetate (1a) (0.2 mmol), benzylamine (2a) (0.24 mmol), I_2_ and K_2_CO_3_ (0.4 mmol), and anhydrous ethyl acetate at 80 °C for 12 h.

**Table 1:** Optimization of the reaction conditions^a^

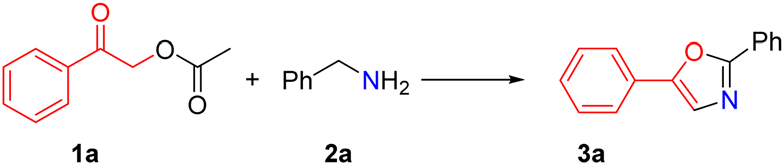			
Entry	Additive	Solvent	3a%^b^
1	K_2_CO_3_/I_2_	Dioxane	Trace
2	K_2_CO_3_/I_2_	Toluene	Trace
3	K_2_CO_3_/I_2_	Chlorobenzene	Trace
4	K_2_CO_3_/I_2_	1,2-Dichloroethane	Trace
5	K_2_CO_3_/I_2_	Acetonitrile	52%
6	K_2_CO_3_/I_2_	*N*,*N*-dimethyl formamide	61%
7	K_2_CO_3_/I_2_	Dimethylsulfoxide	44%
8	K_2_CO_3_/I_2_	Ethyl acetate	90%
9	K_2_CO_3_ (0.4)/I_2_ (0.2)	Ethyl acetate	Trace
	K_2_CO_3_ (0.4)/I_2_ (0.3)		32%
	K_2_CO_3_ (0.4)/I_2_ (0.4)		92%
10	K_2_CO_3_(0.2)/I_2_ (0.4)	Ethyl acetate	Trace
	K_2_CO_3_ (0.3)/I_2_ (0.4)		40%
	K_2_CO_3_ (0.4)/I_2_ (0.4)		92%
11	K_2_CO_3_	Ethyl acetate	Trace
12	I_2_	Ethyl acetate	Trace
13^c^	K_2_CO_3_/I_2_	Ethyl acetate	—
14^d^	K_2_CO_3_/I_2_	Ethyl acetate	35%
15^e^	K_2_CO_3_/I_2_	Ethyl acetate	78%
16	Cs_2_CO_3_/I_2_	Ethyl acetate	42%
17	KOH/I_2_	Ethyl acetate	31%
18	DBU/I_2_	Ethyl acetate	Trace

a Reaction conditions: 2-oxo-2-phenylethyl acetate (1a) (0.2 mmol), benzylamine (2a) (0.24 mmol), I_2_ (0.4 mmol), K_2_CO_3_ (0.4 mmol), solvent (2 mL), N_2_ in 25 mL Schlenk tube, 80 °C, 8 h.

b Isolated yield.

c 25 °C.

d 50 °C.

e 100 °C.

With the optimal reaction conditions established, we proceeded to investigate the substrate scope of the reaction by employing 2-oxo-2-phenylethyl acetate (1a) with a series of electronically diversified benzylamine and other primary amines (2a–2m), the results are shown in Table 2. It was found that benzylamine tolerates a wide range of functionalities, electron donating (CH_3_ and OCH_3_) and withdrawing groups (NO_2_, Br, and Cl), with 2-oxo-2-phenylethyl acetate to afford the corresponding substituted oxazoles (3a–3f). The substrate 2-naphthylmethylamine (2g) was also tolerated in the reaction condition, giving an excellent yield of 3g (85%). Moreover, 2h and 2i bearing a heterocycle moiety were employed as good substrates in the cyclization reaction with 1a, and substituted oxazoles 3h and 3i were isolated in 78% and 81% yields, respectively. In addition, no significant effect was observed for sterically demanding substrates 2j and 2k, which worked well in the reaction with 1a to give desired products 3j and 3k. We were delighted to find that aliphatic amines phenylethylamine and *n*-propylamine could be tolerated under our reaction conditions to give substituted oxazoles 3l and 3m in moderate yields. Thus, the present methodology shows a general applicability in the synthesis of substituted oxazoles.

**Table 2:** Substrate scope of amines^a^

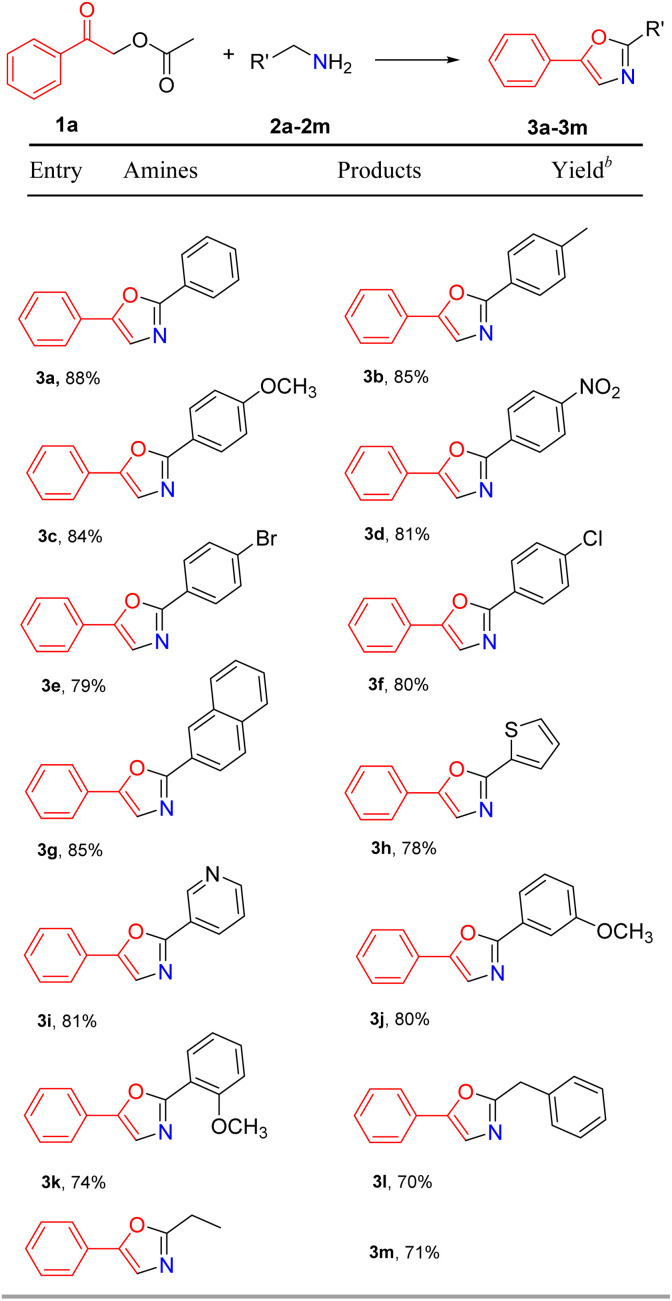

a Reaction conditions: 2-oxo-2-phenylethyl acetate (1a) (0.2 mmol), primary amines (2a–2m) (0.24 mmol), K_2_CO_3_ (0.4 mmol), I_2_ (0.4 mmol) (0.4 mmol), ethyl acetate (2 mL), N_2_ in 10 mL Schlenk tube, 80 °C, and 8 h.

b Isolated yield.

The efficiency of this reaction was further investigated with substituted 2-oxo-2-phenylethyl acetate with benzylamine, as shown in Table 3. It was observed that 2-oxo-2-phenylethyl acetate with electron-rich and electron-deficient substituted groups could be cyclized by benzylamine (2a), giving the corresponding substituted oxazole products in moderate to good yields. For example, the reactions of 2-oxo-2-phenylethyl acetate with electron-rich groups (methyl and methoxy) at the *para*-position on the benzene ring proceeded well to give substituted oxazole products with a yield of 84–88%. Strong electron-deficient substituents such as -nitro, -trifluoromethyl, and -hydroxy could also react smoothly with benzylamine (2a) to give desired target products (3p–3r). It was found that reactions of halogen (F, Br, and Cl) substituted 2-oxo-2-phenylethyl acetate with 2a proceeded well and gave the desired oxazole derivatives 3s, 3t and 3u in 79%, 81% and 80% yields, respectively. Notably, a substituent at the *ortho*- or *meta*-position of the phenyl ring gave the corresponding product 3v and 3w with a yield of 81% and 74%, respectively. These results indicated that the steric effects did not affect the efficiency of the reactions. Interestingly, 2-cyclopropyl-2-oxoethyl acetate was also compatible with the present reaction system to give the desired oxazole product 3x in 69% yield, while alkyl-substituted bromoethyl ketones could not yield corresponding oxazole products in other studies,^15*d*^ indicating that our reaction conditions have wide applicability.1
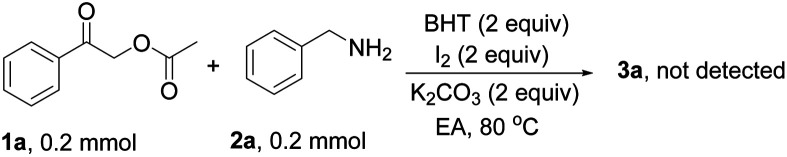
2
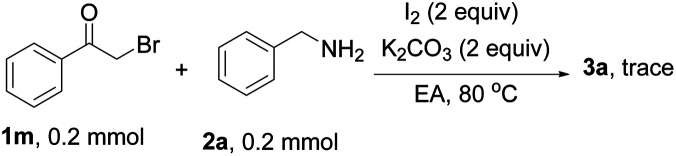
3
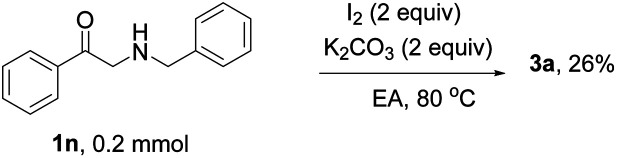


**Table 3:** Substrate scope of substituted 2-oxo-2-phenylethyl acetate^a^

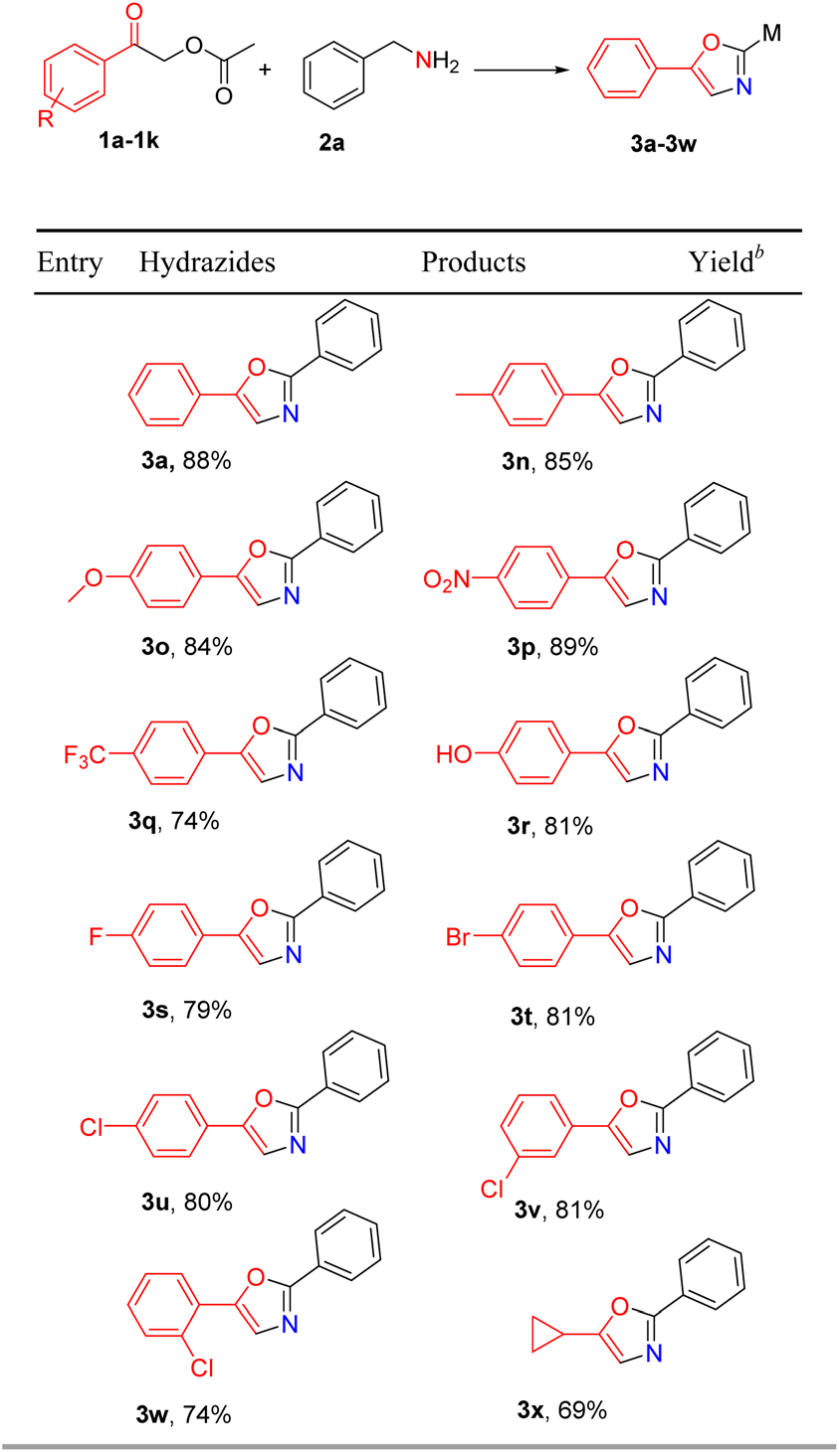

a Reaction conditions: substituted 2-oxo-2-phenylethyl acetate (1a–1l) (0.2 mmol), benzylamine (2a) (0.24 mmol), K_2_CO_3_ (0.4 mmol), I_2_ (0.4 mmol) (0.4 mmol), ethyl acetate (2 mL), N_2_ in 10 mL Schlenk tube, 80 °C, and 8 h.

b Isolated yield.

To get some information on the reaction mechanism, a control experiment was carried out as discussed below. When radical inhibitor 2,6-di-*tert*-butyl-4-methylphenol (BHT) was added to the reaction medium, 3a was not detected, which suggested that a free radical process was involved in the present reaction (eqn (1)). When 2-bromoacetophenone 1m replaced 2-oxo-2-phenylethyl acetate as the substrate, the trace product 3a was detected, suggesting that substituted bromoacetophenone is not an interim process in the present reaction system (eqn (2)). The 2-(benzylamino)-1-phenylethan-1-one 1n was used as a reactive material under the standard condition, 26% 3a was obtained proving that the reaction probably proceeded through imine intermediates (eqn (2)).4
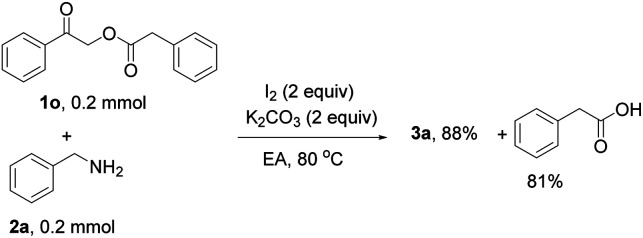


To confirm the formation of by-products during the reaction, 2-oxo-2-phenylethyl 2-phenylacetate (1o) was used as a substrate. 3a was obtained in 88% yield (eqn (4)), and phenylacetic acid was detected in 81% yield, proving that C–O bond cleavage actually occurs in the reaction.

**Scheme 1:**
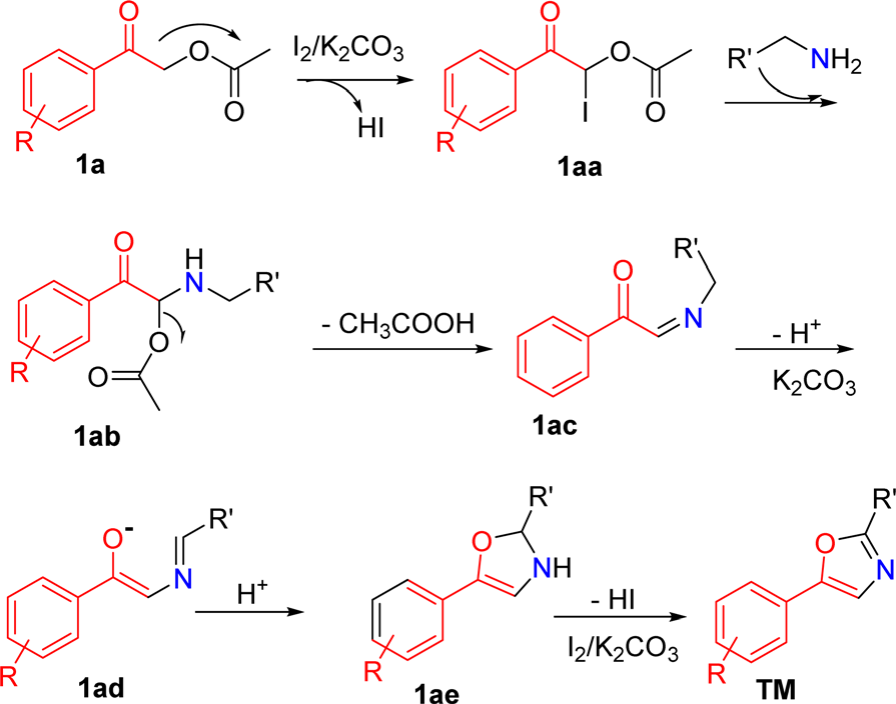
Plausible reaction pathway for the synthesis of substituted oxazoles.

Based on the results described above and those of the previous reports, the possible pathway for the synthesis of substituted oxazoles is given in Scheme 1:^15*a*,*d*^ K_2_CO_3_ promoted iodination to produce iodine intermediate 1aa; the S_N_^2^ reaction between 1aa and amines proceeded to produce the intermediate 1ab; and the decomposition of the alkyl carbonate *via* C–O bond cleavage led to the removal of an α-proton and the release of acetic acid to produce imine intermediate 1ac, followed by enolization of 1ac to obtain 1ad, intramolecular nucleophilic addition to produce 1ae, and iodine oxidation of 1ae to obtain substituted oxazole TM. Although 4 equiv. of acid was produced theoretically, 2 equiv. of K_2_CO_3_ was sufficient because its conjugated acid (HCO_3_^−^) was involved in the deprotonation process.

## Conclusion

In summary, a strategy for the metal-free C–O bond cleavage of ester and functionalization for the synthesis of substituted oxazoles has been developed. C–O bond cleavage together with C–N and C–O bond formation are realized in one pot *via* iodine as the sole oxidant. The present findings not only provide a general and concise method for the preparation of substituted oxazoles but also open an avenue for the selective C–O bond cleavage of esters.

## Conflicts of interest

There are no conflicts to declare.

## Supplementary Material

RA-014-D4RA05122J-s001
