# Supplementary material for: Metal free C–O bond cleavage: a new strategy for the synthesis of substituted oxazoles
Source: RSC Adv. 2024 Sep 4;14(38):28210–4. doi: 10.1039/d4ra05122j (PMC11372780; doi:10.1039/d4ra05122j)
Supplement: RA-014-D4RA05122J-s001 [file RA-014-D4RA05122J-s001.pdf]

Supplementary Information (SI) for RSC Advances.
This journal is © The Royal Society of Chemistry 2024

Supporting Information

Metal free C−O bond cleavage: a new strategy for the synthesis of substituted oxazoles

Shengwang Li,[a] Guiqin Liu,[a] Zheyan Zhang,[a] Ruiling, Chen,[b]* Haiying Tian,[b]* Huifeng Wang,[a]* Xiuling Chen[a]*

[a]Hubei Key Laboratory of Radiation Chemistry and Functional Materials, Hubei University of Science and Technology, Xianning 437100, China

[b]School of Pharmacy, Changzhi Medical College, Changzhi, 046000, China

[Fax: (+86)-715-8338072; E-mail: cxl828800@163.com (Dr. X. L. Chen)]

Contents

General remarks	2
General procedure	2
^{1}H and ^{13}C NMR data of products	3-9
References	9-10
Copies of ^{1}H and ^{13}C NMR spectra	11-34

1. General remarks

All non-aqueous reactions and manipulations were performed in air atmosphere. All solvents were purchased from Energy Chemical, Aladdin and used without further treatment. The reactions were monitored by GC (7820A, Hubei University of Science and Technology) and GC-MS (QP2010, Hunan University). The electron ionization (EI) method was used as the ionization method for the HRMS measurement, and the mass analyzer type is TOF for EI. The ^{1}H NMR and ^{13}C NMR spectra were recorded on a Brucker ADVANCE III spectrometer at 400 MHz and 100 MHz, respectively (Hubei University of Science and Technology). Flash column chromatography was performed using silica gel 40-70 μm (200-300 mu). Amines and azide compounds were purchased from Energy Chemical and used without further treatment.

2. General procedure

A 10-mL Schlenk-type tube equipped with a magnetic stir bar was charged with substituted 2-oxo-2-phenylethyl acetate **1a-1l** (0.2 mmol), primary amine **2a-2m** (0.24 mmol), K_2CO_3 (0.4 mmol), I_2 (0.4 mmol), under N_2 atmospheres, ethyl acetate (2 mL) was added at room temperature, and then the reaction mixture was stirred at 80 °C for 8 h, the reaction was monitored by GC or GC-MS. After completion of the reaction, the resulting solution was cooled to room temperature, and neutralized with saturated NaCl solution. The product was extracted with ethyl acetate, dried over anhydrous Mg_2SO_4 and concentrated in vacuum. The crude product was purified by flash column chromatography on silica gel and eluted with ethyl acetate/Petroleum ether (1/5-1/10) give analytically pure product.

Table S1 Optimization of the model reaction condition of bases[a]

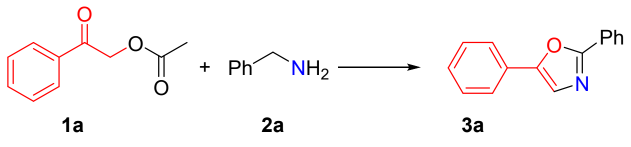

Entry	Additive	Solvent	**3a**%[b]
1	$NaOH/I_2$	ethyl acetate	35%
2	KOH/I_2	ethyl acetate	47%
3	NEt_3/I_2	ethyl acetate	trace

4	Na_2CO_3/I_2	ethyl acetate	63%
5	Cs_2CO_3/I_2	ethyl acetate	52%
6	C_4H_9OK /I_2	ethyl acetate	81%
7	K_2CO_3/I_2	ethyl acetate	90%

[a]Reaction conditions: 2-oxo-2-phenylethyl acetate **1a** (0.2 mmol), benzylamine **2a** (0.24 mmol), I_2 (0.4 mmol), base (0.4 mmol), ethyl acetate (2 mL), N_2 in 25 mL schlenk tube, 80 ^oC, 8 h. [b]Isolated yield.

3. ^1H NMR and ^{13}C NMR data of products

2,5-diphenyloxazole (3a)[1]

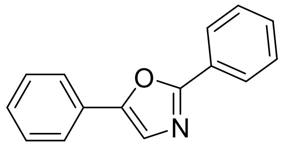

Following the general procedure (EtOAc/Petroleum ether 1:20), **3a** was obtained as a colorless solid, isolated yield: 88%, m.p. 66-68 ^oC. ^1H NMR ($CDCl_3$, 400 MHz): δ 8.12 (q, 2H, J = 4.0 Hz), 7.71 (d, 2H, J = 7.6 Hz), 7.42-7.50 (m, 6H), 7.34 (t, 1H, J = 7.4 Hz); ^{13}C NMR ($CDCl_3$, 100 MHz): δ 161.2, 151.3, 130.4, 128.9, 128.8, 128.5, 128.1, 127.5, 126.3, 124.2, 123.5; GC-MS: m/z = 221.25.

5-phenyl-2-(p-tolyl)oxazole (3b)[2]

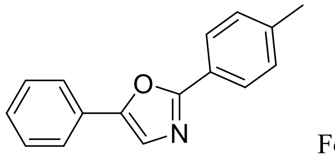

Following the general procedure (EtOAc/Petroleum ether 1:5), **3b** was obtained as a colorless solid, isolated yield: 85%, m.p. 71-73 ^oC. ^1H NMR ($CDCl_3$, 400 MHz): δ 8.00 (d, 2H, J = 8.0 Hz), 7.71 (d, 2H, J = 7.6 Hz), 7.44 (t, 3H, J = 7.0 Hz), 7.33 (d, 1H, J = 7.6 Hz), 7.28 (d, 2H, J = 8.0 Hz), 2.41 (s, 3H); ^{13}C NMR ($CDCl_3$, 100 MHz): δ 161.1, 151.0, 140.7, 129.6, 128.9, 128.3, 128.2, 126.3, 124.8, 124.2, 123.4, 21.6; GC-MS: m/z = 235.09.

2-(4-methoxyphenyl)-5-phenyloxazole (3c)[1]

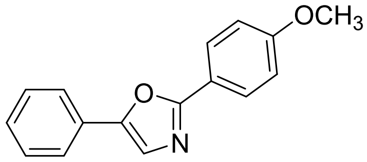

Following the general procedure (EtOAc/Petroleum ether 1:5), **3c** was obtained as a colorless solid, isolated yield: 84%, m.p. 78-79 ^oC. ^1H NMR ($CDCl_3$, 400 MHz): δ 7.71 (t, 3H, J = 6.6 Hz), 7.64 (s, 1H), 7.34-7.46 (m, 5H), 7.00 (q, 1H, J = 4.2 Hz), 3.89 (s, 3H); ^{13}C NMR ($CDCl_3$, 100 MHz): δ 161.1, 159.9, 151.3, 129.9, 128.9, 128.7, 128.5, 128.0, 124.2, 123.4, 118.8, 116.9, 111.0, 55.5; GC-MS: m/z = 251.09.

2-(4-nitrophenyl)-5-phenyloxazole (3d)[3]

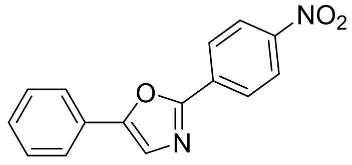

Following the general procedure (EtOAc/Petroleum ether 1:5), **3d** was obtained as a colorless solid, isolated yield: 81%, m.p. 209-213 °C. ^{1}H NMR ($CDCl_3$, 400 MHz): δ 8.34 (d, 2H, *J* = 9.2 Hz), 8.13-8.15 (m, 2H), 7.86 (d, 2H, *J* = 8.8 Hz), 7.66 (s, 1H), 7.52 (t, 3H, *J* = 3.2 Hz); ^{13}C NMR ($CDCl_3$, 100 MHz): δ 162.8, 149.1, 147.2, 133.8, 131.1, 129.0, 126.9, 126.8, 126.7, 124.6, 124.5; GC-MS: m/z = 266.25.

2-(4-bromophenyl)-5-phenyloxazole (3e)[4]

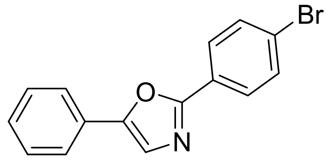

Following the general procedure (EtOAc/Petroleum ether 1:5), **3e** was obtained as a colorless solid, isolated yield: 79%, m.p. 100-102 °C. ^{1}H NMR ($CDCl_3$, 400 MHz): δ 7.94 (d, 2H, *J* = 8.8 Hz), 7.69 (d, 2H, *J* = 7.6 Hz), 7.69 (d, 2H, *J* = 8.8 Hz), 7.41-7.45 (m, 3H), 7,34 (t, 1H, *J* = 7.4 Hz); ^{13}C NMR ($CDCl_3$, 100 MHz): δ 171.1, 160.3, 151.6, 132.1, 128.9, 128.6, 127.8, 127.7, 126.4, 124.8, 124.2, 123.6; GC-MS: m/z = 298.99.

2-(4-chlorophenyl)-5-phenyloxazole (3f)[5]

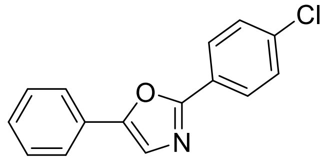

Following the general procedure (EtOAc/Petroleum ether 1:5), **3f** was obtained as a colorless solid, isolated yield: 80%, m.p. 103-105 °C. ^{1}H NMR ($CDCl_3$, 400 MHz): δ 8.04 (d, 2H, *J* = 8.8 Hz), 7.71 (d, 2H, *J* = 7.6 Hz), 7.42-7.56 (m, 5H), 7.34 (t, 1H, *J* = 7.4 Hz); ^{13}C NMR ($CDCl_3$, 100 MHz): δ 160.2, 151.6, 136.4, 129.1, 128.9, 128.6, 127.9, 127.5, 125.9, 124.3, 123.6; GC-MS: m/z = 255.04.

2-(naphthalen-2-yl)-5-phenyloxazole (3g)[2]

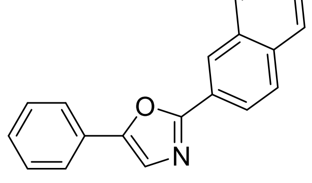

Following the general procedure (EtOAc/Petroleum ether 1:5), **3i** was obtained as a colorless solid, isolated yield: 85%, m.p. 99-102 °C. ^{1}H NMR ($CDCl_3$, 400 MHz): δ 8.60 (s, 1H),8.19 (d, 1H, *J* = 8.4 Hz), 7.77-7.93 (m, 5H), 7.47-7.56 (m, 5H), 7.38 (t, 1H, *J* = 7.2

Hz); ^{13}C NMR ($CDCl_3$, 100 MHz): δ 161.4, 151.5, 134.2, 133.1, 128.9, 128.73, 128.71, 128.5, 128.1, 127.9, 127.3, 126.8, 126.2, 124.8, 124.3, 123.6, 123.2; GC-MS: m/z = 271.09.

5-phenyl-2-(thiophen-2-yl)oxazole (3h)[5]

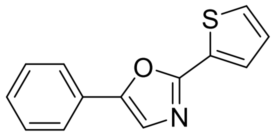

Following the general procedure (EtOAc/Petroleum ether 1:5), **3g** was obtained as a colorless solid, isolated yield: 78%, m.p. 72-74 °C. ^{1}H NMR ($CDCl_3$, 400 MHz): δ 7.74 (d, 1H, J = 3.6 Hz), 7.71 (d, 2H, J = 7.6 Hz), 7.42-7.46 (m, 3H), 7.36 (s, 1H), 7.34 (t, 1H, J = 7.4 Hz), 7.14 (q, 1H, J = 4.4 Hz); ^{13}C NMR ($CDCl_3$, 100 MHz): δ 157.4, 150.9, 130.1, 128.9, 128.5, 128.3, 127.9, 127.8, 127.6, 124.2, 123.3; GC-MS: m/z = 227.04.

5-phenyl-2-(pyridin-3-yl)oxazole (3i)[1]

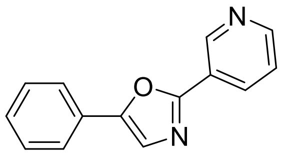

Following the general procedure (EtOAc/Petroleum ether 1:5), **3h** was obtained as a colorless solid, isolated yield: 81%, m.p. 77-79 °C. ^{1}H NMR ($CDCl_3$, 400 MHz): δ 9.35 (d, 1H, J = 1.2 Hz), 8.69 (d, 1H, J = 3.2 Hz), 8.36 (d, 1H, J = 8.0 Hz), 7.73 (d, 2H, J = 7.6 Hz), 7.36-7.49 (m, 5H); ^{13}C NMR ($CDCl_3$, 100 MHz): δ 158.7, 152.1, 150.9, 147.5, 133.5, 129.0, 128.9, 127.6, 124.4, 123.8, 123.7; GC-MS: m/z = 238.11.

2-(3-methoxyphenyl)-5-phenyloxazole (3j)[6]

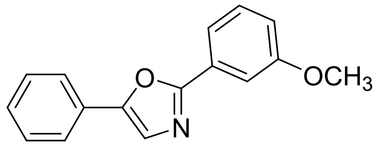

Following the general procedure (EtOAc/Petroleum ether 1:5), **3j** was obtained as a colorless solid, isolated yield: 80%, m.p. 81-84 °C. ^{1}H NMR ($CDCl_3$, 400 MHz): δ 7.71 (t, 2H, J = 6.6 Hz), 7.64 (s, 1H), 7.34-7.46 (m, 5H), 7.00 (q, 1H, J = 8.4 Hz); ^{13}C NMR ($CDCl_3$, 100 MHz): δ 161.1, 159.9, 151.3, 129.9, 128.9, 128.7, 128.5, 128.0, 124.2, 123.4, 118.8, 116.9, 111.0, 55.5; GC-MS: m/z = 251.09.

diphenyl benzylphosphoramidate (3k)[3]

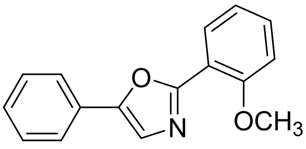 Following the general procedure (EtOAc/Petroleum ether 1:5), **3j** was obtained as a colorless solid, isolated yield: 74%, m.p. 77-79 °C. ^{1}H NMR ($CDCl_3$, 400 MHz): δ 7.74 (t, 2H, *J* = 6.6 Hz), 7.65 (s, 1H), 7.33-7.47 (m, 5H), 7.01 (q, 1H, *J* = 8.4 Hz); ^{13}C NMR ($CDCl_3$, 100 MHz): δ 161.1, 159.9, 151.4, 130.0, 129.0, 128.7, 128.5, 128.0, 124.3, 123.5, 118.8, 116.9, 111.0, 55.5; GC-MS: m/z = 251.09.

2-benzyl-5-phenyloxazole (3l)[2]

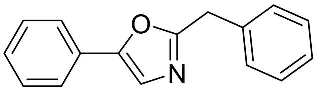 Following the general procedure (EtOAc/Petroleum ether 1:10), **3l** was obtained as a colorless solid, isolated yield: 70%, m.p. 71-72C. ^{1}H NMR ($CDCl_3$, 400 MHz): δ 8.31 (d, 2H, *J* = 7.2 Hz), 7.47 (t, 2H, *J* = 7.6 Hz), 7.24-7.49 (m, 7H), 4.18 (s, 2H); ^{13}C NMR ($CDCl_3$, 100 MHz): δ 167.7, 138.3, 125.5, 134.4, 131.2, 128.83, 128.75, 127.1, 126.8, 124.1, 122.0, 40.6; GC-MS: m/z = 235.09.

2-ethyl-5-phenyloxazole (3m)[1]

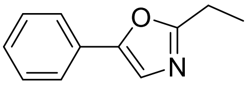 Following the general procedure (EtOAc/Petroleum ether 1:10), **3m** was obtained as a colorless liquid, isolated yield: 71%. ^{1}H NMR ($CDCl_3$, 400 MHz): δ 7.75 (d, 2H, *J* = 7.2 Hz), 7.34 (t, 2H, *J* = 7.6 Hz), 7.19 (t, 1H, *J* = 7.4 Hz), 7.09 (s, 1H); ^{13}C NMR ($CDCl_3$, 100 MHz): δ 149.7, 139.9, 134.5, 128.5, 126.3, 124.7, 114.7, 20.3, 11.2; GC-MS: m/z = 173.08.

2-phenyl-5-(p-tolyl)oxazole (3n)[1]

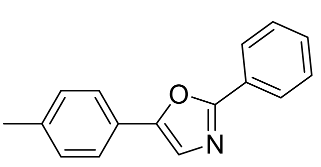 Following the general procedure (EtOAc/Petroleum ether 1:10), **3n** was obtained as a colorless solid, isolated yield: 85%, m.p. 69-71 °C. ^{1}H NMR ($CDCl_3$, 400 MHz): δ 8.12 (d, 2H, *J* = 8.0 Hz), 7.60 (d, 2H, *J* = 8.0 Hz), 7.45-7.50 (m, 3H), 7.39 (s, 1H), 7.24 (d, 2H, *J* = 7.6 Hz), 2,39 (s, 3H); ^{13}C NMR ($CDCl_3$, 100 MHz): δ 160.9, 151.5, 138.5, 130.2, 129.6, 128.8, 127.6, 126.2, 125.3, 124.2, 122.8, 21.4; GC-MS: m/z = 235.09.

5-(4-methoxyphenyl)-2-phenyloxazole (3o)[1]

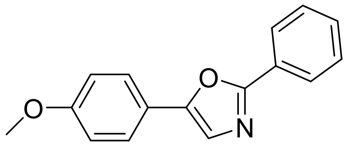

Following the general procedure (EtOAc/Petroleum ether 1:10), **3o** was obtained as a colorless solid, isolated yield: 84%, m.p. 88-89 ^{o}C. ^{1}H (CDCl$_3$, 400 MHz): δ 8.10 (d, 2H, *J* = 6.4 Hz), 7.64 (d, 2H, *J* = 8.8 Hz), 7.42-7.49 (m, 3H), 7.32 (s, 1H), 6.97 (d, 2H, *J* = 8.8 Hz), 3.86 (s, 3H); ^{13}C NMR (CDCl$_3$, 100 MHz): δ 160.6, 159.9, 151.4, 130.1, 128.8, 127.7, 126.2, 125.8, 122.0, 121.0, 114.5, 55.4; GC-MS: m/z = 251.09.

5-(4-nitrophenyl)-2-phenyloxazole (3p)[7]

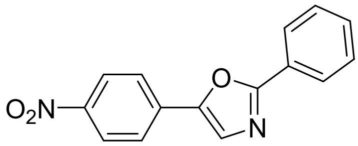

Following the general procedure (EtOAc/Petroleum ether 1:10), **3p** was obtained as a colorless solid, isolated yield: 89%, m.p. 112-115 ^{o}C. ^{1}H NMR (CDCl$_3$, 400 MHz): δ 8.31 (d, 2H, *J* = 9.2 Hz), 8.13-8.15 (m, 2H), 7.86 (d, 2H, *J* = 8.8 Hz), 7.66 (s, 1H), 7.52 (d, 3H, *J* = 3.2 Hz); ^{13}C NMR (CDCl$_3$, 100 MHz): δ 162.8, 149.1, 147.2, 133.8, 131.1, 129.0, 126.9, 126.8, 126.7, 124.6, 124.5; GC-MS: m/z = 266.06.

2-phenyl-5-(4-(trifluoromethyl)phenyl)oxazole (3q)[1]

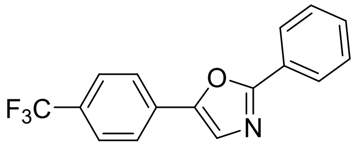

Following the general procedure (EtOAc/Petroleum ether 1:10), **3q** was obtained as a colorless solid, isolated yield: 74%, m.p. 203-205 ^{o}C. ^{1}H NMR (CDCl$_3$, 400 MHz): δ 8.12 (q, 2H, *J* = 3.8 Hz), 7.81 (d, 2H, *J* = 8.0 Hz), 7.69 (d, 2H, *J* = 8.0 Hz), 7.56 (s, 1H), 7.49 (q, 3H, *J* = 2.4 Hz); ^{13}C NMR (CDCl$_3$, 100 MHz): δ 162.0, 149.9, 131.6, 131.2 (d, $J_{c\text{-}f}$= 94 Hz), 130.8, 129.9 128.9, 127.1, 126.5, 126.0 (d, $J_{c\text{-}f}$ = 4.0 Hz), 125.2, 124.2; GC-MS: m/z = 289.07.

4-(2-phenyloxazol-5-yl)phenol (3r)[6]

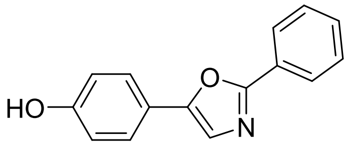

Following the general procedure (EtOAc/Petroleum ether 1:10), **3r** was obtained as a colorless solid, isolated yield: 81%, m.p. > 250 ^{o}C. ^{1}H NMR (DMSO-*d*, 400 MHz): δ 9.89 (s, 1H), 8.06 (t, 2H, *J* = 6.4 Hz), 7.65 (d, 2H, *J* = 8.8 Hz), 7.59 (s, 1H), 7.49-7.57 (m, 3H), 6.87 (d, 2H, *J* = 8.4 Hz); ^{13}C NMR (DMSO-*d*, 100 MHz): δ 159.7, 158.5, 151.8, 130.8, 129.6, 127.5, 126.3, 126.2, 122.3, 119.0, 116.4; GC-MS: m/z = 237.07.

5-(4-fluorophenyl)-2-phenyloxazole (3s)[1]

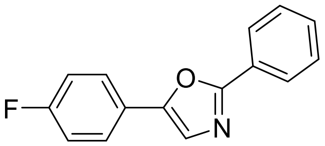

Following the general procedure (EtOAc/Petroleum ether 1:10), **3s** was obtained as a colorless solid, isolated yield: 79%, m.p. 72-74 °C. ^{1}H NMR ($CDCl_3$, 400 MHz): δ 8.09 (q, 2H, J = 7.2 Hz), 7.69-7.72 (m, 2H), 7.47-7.49 (m, 3H), 7.38 (s, 1H), 7.15 (d, 2H, J = 8.8 Hz); ^{13}C NMR ($CDCl_3$, 100 MHz): δ 161.2 (d, $J_{c\text{-}f}$= 30 Hz), 150.5, 130.4, 129.1, 128.9, 129.9, 127.2, 126.3, 126.1 (d, $J_{c\text{-}f}$= 8.0 Hz), 123.1, 116.0 (d, $J_{c\text{-}f}$= 22 Hz); GC-MS: m/z = 239.07.
从

5-(4-bromophenyl)-2-phenyloxazole (3t)[6]

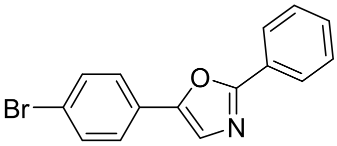

Following the general procedure (EtOAc/Petroleum ether 1:10), **3t** was obtained as a colorless solid, isolated yield: 81%, m.p. 101-103 °C. ^{1}H NMR ($CDCl_3$, 400 MHz): δ 8.09 (q, 2H, J = 4.0 Hz), 7.58 (s, 4H), 7.45-7.49 (m, 4H); ^{13}C NMR ($CDCl_3$, 100 MHz): δ 161.5, 150.3, 132.2, 130.5, 128.9, 127.3, 127.0, 126.4, 125.7, 124.0, 123.3; GC-MS: m/z = 298.99.

5-(4-chlorophenyl)-2-phenyloxazole (3u)[1]

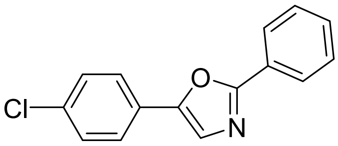

Following the general procedure (EtOAc/Petroleum ether 1:10), **3u** was obtained as a colorless solid, isolated yield: 80%, m.p. 110-112 °C. ^{1}H NMR ($CDCl_3$, 400 MHz): δ 8.09 (q, 2H, J = 3.8 Hz), 7.62 (d, 2H, J = 8.4 Hz), 7.46-7.50 (m, 3H), 7.41 (d, 2H, J = 3.6 Hz), 7.39 (s, 1H); ^{13}C NMR ($CDCl_3$, 100 MHz): δ 161.4, 150.3, 134.2, 130.5, 129.2, 128.9, 127.3, 126.5, 126.4, 125.4, 123.9; GC-MS: m/z = 255.04.

5-(3-chlorophenyl)-2-phenyloxazole (3v)[4]

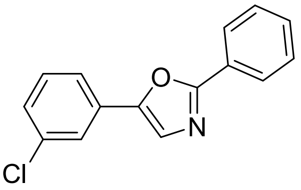

Following the general procedure (EtOAc/Petroleum ether 1:10), **3v** was obtained as a colorless solid, isolated yield: 81%, 102-104 °C. ^{1}H NMR ($CDCl_3$, 400 MHz): δ 8.11 (m, 2H), 7.69 (s, 1H), 7.57 (d, 1H, J = 8.0 Hz), 7.46-7.49 (m, 4H), 7.36 (t, 1H, J = 8.0 Hz), 7.28 (d, 1H, J = 8.4 Hz); ^{13}C NMR ($CDCl_3$, 100 MHz): δ 161.6, 149.9, 135.1, 130.6, 130.2, 129.7, 128.9, 128.4, 127.2, 126.4, 124.4, 124.2, 122.2; GC-MS: m/z = 255.04

5-(2-chlorophenyl)-2-phenyloxazole (3w)[7]

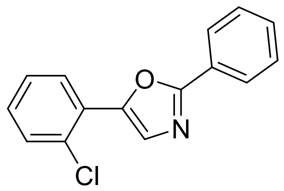

Following the general procedure (EtOAc/Petroleum ether 1:10), **3w** was obtained as a colorless solid, isolated yield: 74%, 101-102 ^{o}C. ^{1}H NMR ($CDCl_3$, 400 MHz): δ 8.12 (q, 2H, J = 3.8 Hz), 7.72 (s, 1H), 7.59 (d, 1H, J = 8.0 Hz), 7.48-7.51 (m, 4H), 7.36 (t, 1H, J = 8.0 Hz), 7.31 (d, 1H, J = 8.4 Hz); ^{13}C NMR ($CDCl_3$, 100 MHz): δ 161.6, 149.9, 135.1, 130.6, 130.3, 129.7, 128.9, 128.4, 127.3, 126.4, 124.5, 124.2, 122.3; GC-MS: m/z = 255.04.

5-cyclopropyl-2-phenyloxazole (3x)[4]

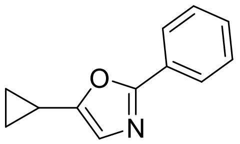

Following the general procedure (EtOAc/Petroleum ether 1:10), **3x** was obtained as a colorless solid, isolated yield: 69%, 70-72 ^{o}C. ^{1}H NMR ($CDCl_3$, 400 MHz): δ 7.99 (q, 2H, J = 3.8 Hz), 7.40-7.45 (m, 3H), 6.81 (s, 1H), 1.92-1.99 (m, 1H), 0.97-1.00 (m, 2H), 0.85-0.88 (m, 2H); ^{13}C NMR ($CDCl_3$, 100 MHz): δ 161.1, 154.6, 129.9, 128.7, 127.8, 126.0, 122.6, 6.7, 6.5; GC-MS: m/z = 185.08.

4. References

(1) Chatterjee, T.; Cho, J. Y.; Cho, E. J. Synthesis of Substituted Oxazoles by Visible-Light Photocatalysis. *J. Org. Chem.* **2016**, *81* (16), 6995–7000. https://doi.org/10.1021/acs.joc.6b00989.

(2) Xu, Z.; Zhang, C.; Jiao, N. Synthesis of Oxazoles through Copper-Mediated Aerobic Oxidative Dehydrogenative Annulation and Oxygenation of Aldehydes and Amines. *Angew. Chem. Int. Ed.* **2012**, *51* (45), 11367–11370. https://doi.org/10.1002/anie.201206382.

(3) Yuan, S.; Li, Z.; Xu, L. Convenient One-Pot Synthesis of 2,5-Disubstituted Oxazoles via a Catalytic Oxidative Dehydrogenation of $F_3CSO_3H\cdot SiO_2$ -DDQ/$CuCl_2$ /LiCl: Oxazole, Aldoximes, Cyclization, DDQ, Catalytic Oxidation. *J. Heterocycl. Chem.* **2013**, *50* (6), 1405–1409. https://doi.org/10.1002/jhet.1637.

(4) Yu, X.; Chen, K.; Wang, Q.; Zhang, W.; Zhu, J. Synthesis of 2,5-Disubstituted Oxazoles *via* Cobalt(III)-Catalyzed Cross-Coupling of *N* -Pivaloyloxyamides and Alkynes. *Chem. Commun.* **2018**, *54* (10), 1197–1200. https://doi.org/10.1039/C7CC08611C.

(5) Zhang, X.; He, Y.; Li, J.; Wang, R.; Gu, L.; Li, G. CO_2 /Photoredox-Cocatalyzed Tandem Oxidative Cyclization of α-Bromo Ketones and Amines To Construct Substituted Oxazoles. *J. Org. Chem.* **2019**, *84* (12), 8225–8231. https://doi.org/10.1021/acs.joc.9b00283.

(6) Jiang, H.; Huang, H.; Cao, H.; Qi, C. TBHP/I $_2$ -Mediated Domino Oxidative Cyclization for One-Pot Synthesis of Polysubstituted Oxazoles. *Org. Lett.* **2010**, *12* (23), 5561–5563. https://doi.org/10.1021/ol1023085.

(7) Hu, T.; Yan, H.; Liu, X.; Wu, C.; Fan, Y.; Huang, J.; Huang, G. Metal-Free Sp3 C–H Functionalization: PABS/I2-Promoted Synthesis of Polysubstituted Oxazole Derivatives from Arylethanones and 2-Amino-2-Alkyl/Arylacetic Acid. *Synlett* **2015**, *26* (20), 2866–2869. https://doi.org/10.1055/s-0035-1560660.

Copies of ^{1}H NMR and ^{13}C NMR spectra

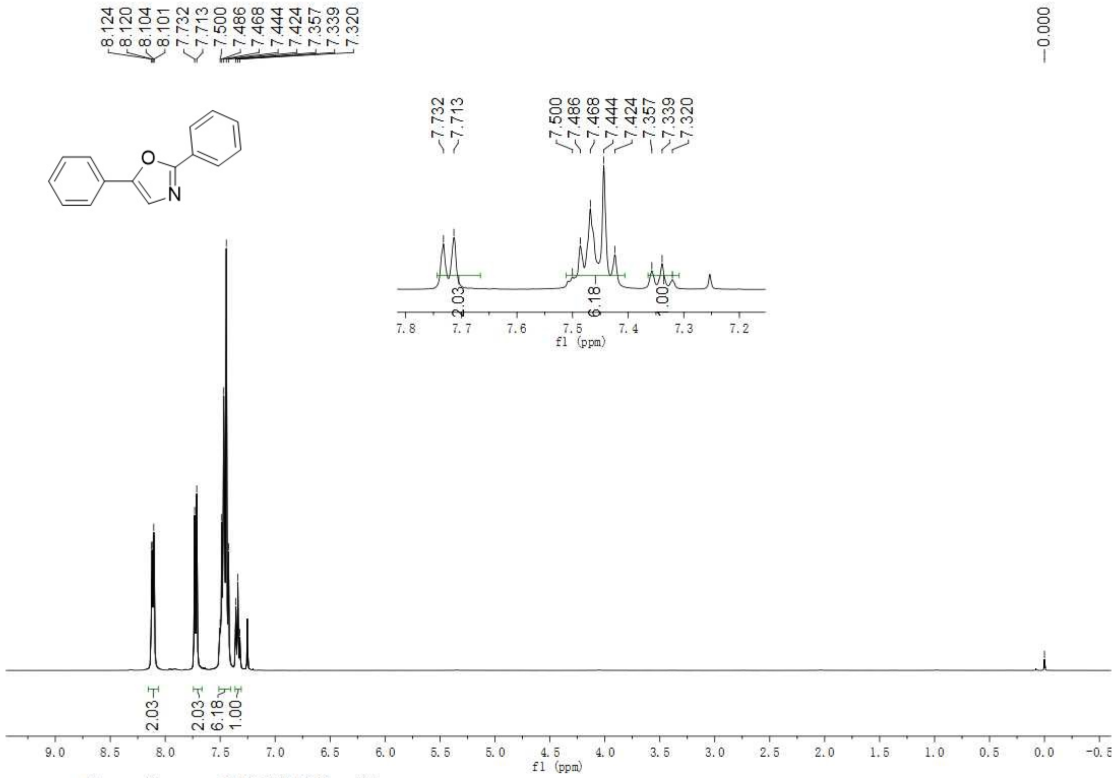

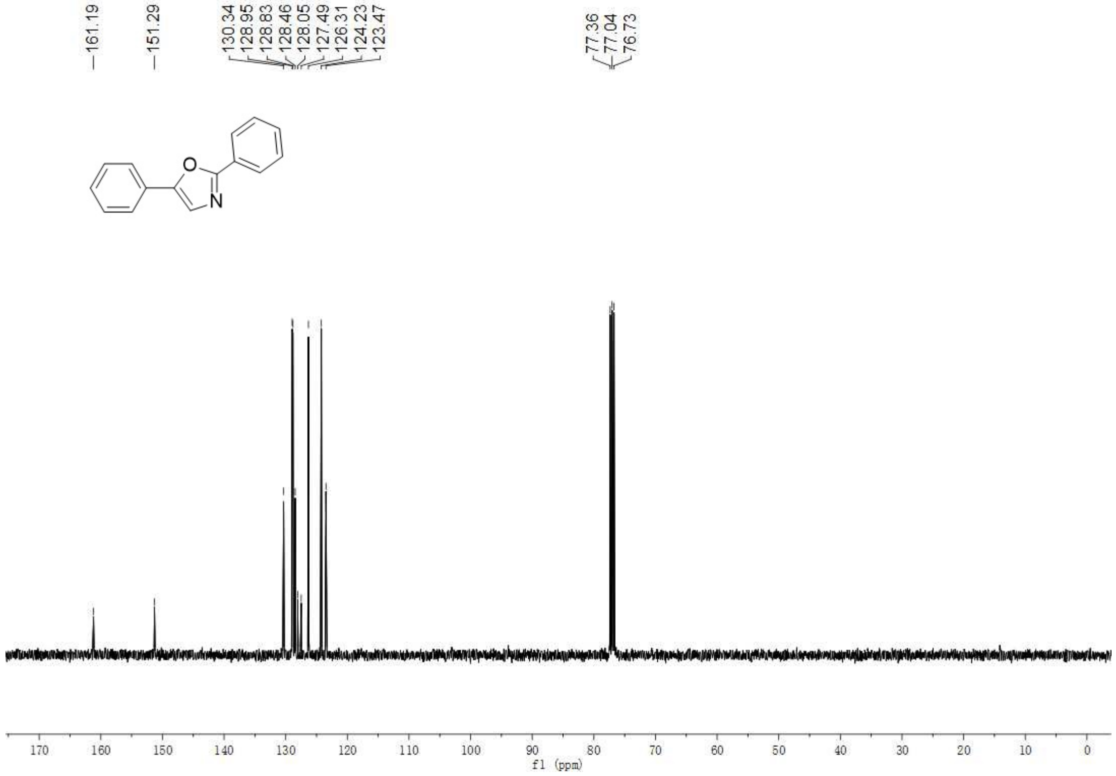

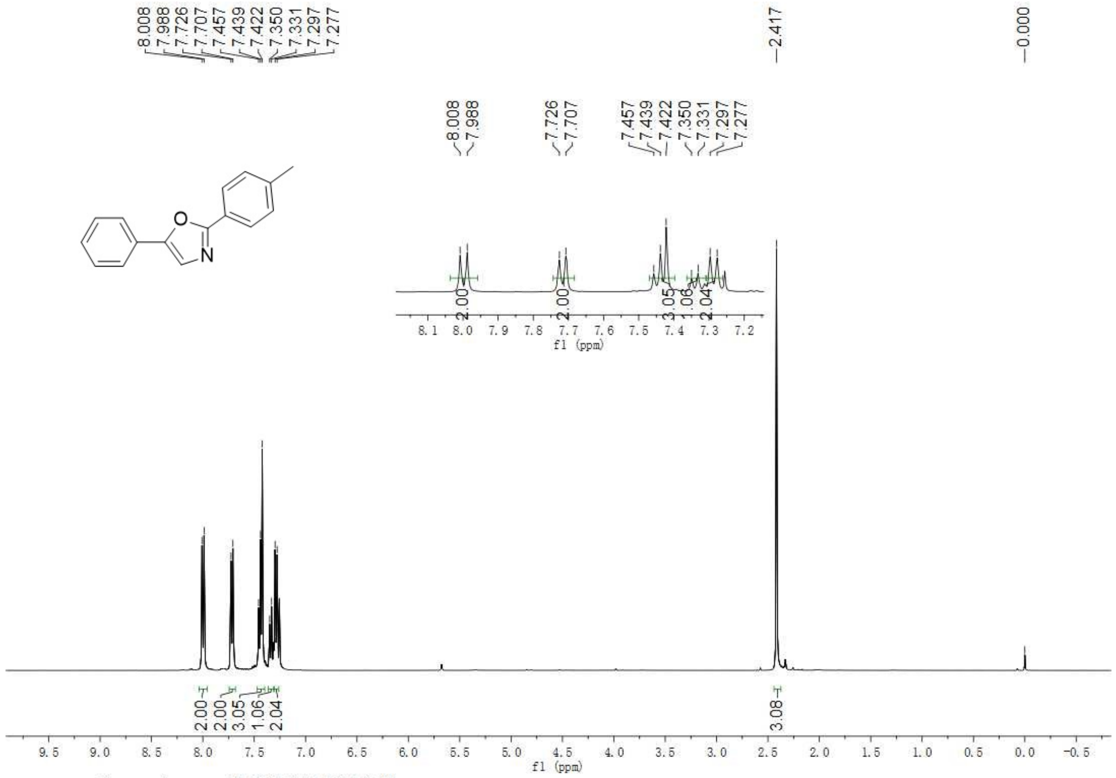
8.008
7.988
7.726
7.707
7.457
7.439
7.422
7.350
7.331
7.297
7.277
2.417
0.000
O
N
2.00
2.00
3.05
1.06
2.04
3.08
f1 (ppm)

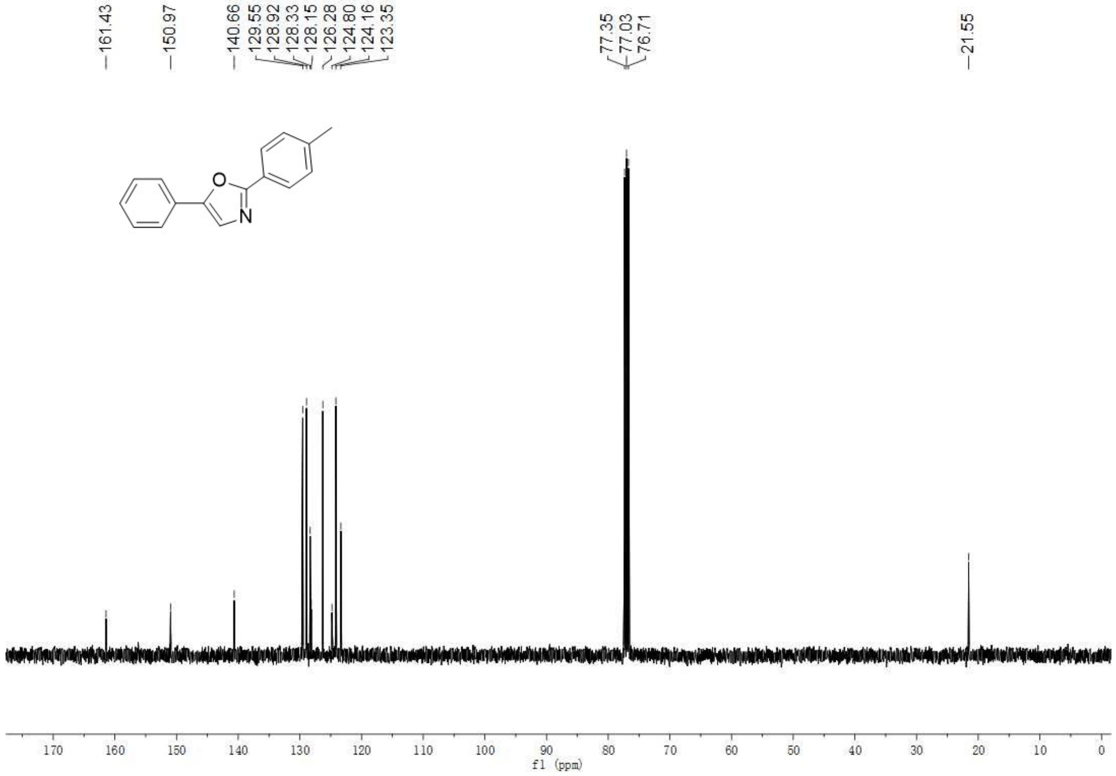
161.43
150.97
140.66
129.55
128.92
128.33
128.15
126.28
124.80
124.16
123.35
77.35
77.03
76.71
21.55
O
N
f1 (ppm)

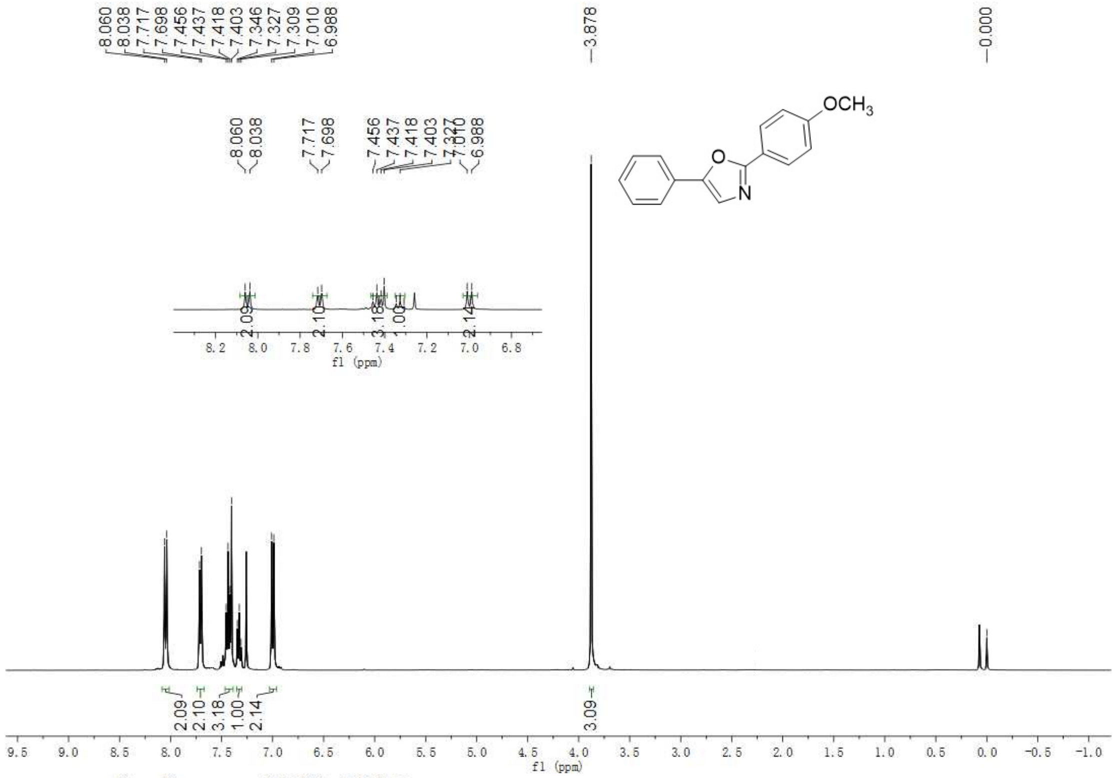
8.060
8.038
7.717
7.698
7.456
7.437
7.418
7.403
7.346
7.327
7.309
7.010
6.988
3.878
0.000
OCH3
O
N
2.09
2.10
3.18
1.00
2.14
3.09
f1 (ppm)

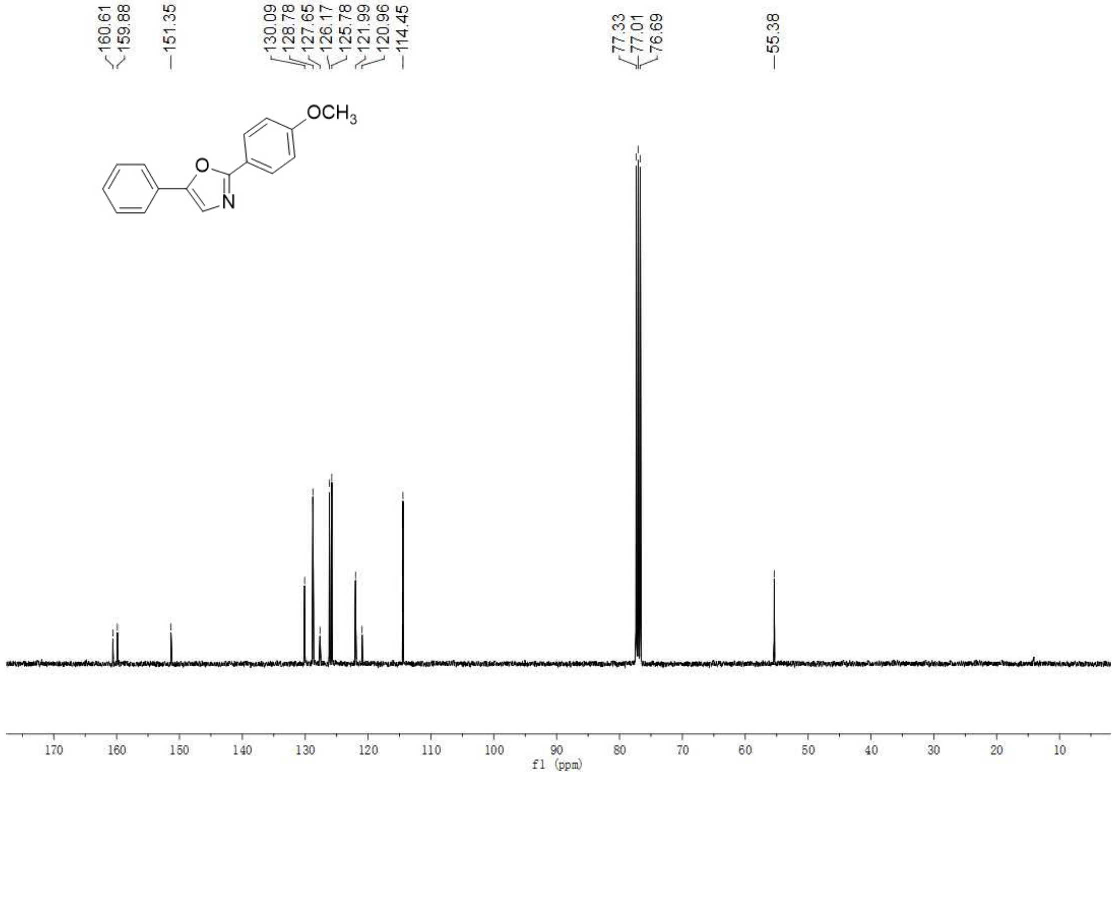
160.61
159.88
151.35
130.09
128.78
127.65
126.17
125.78
121.99
120.96
114.45
77.33
77.01
76.69
55.38
OCH3
O
N
f1 (ppm)

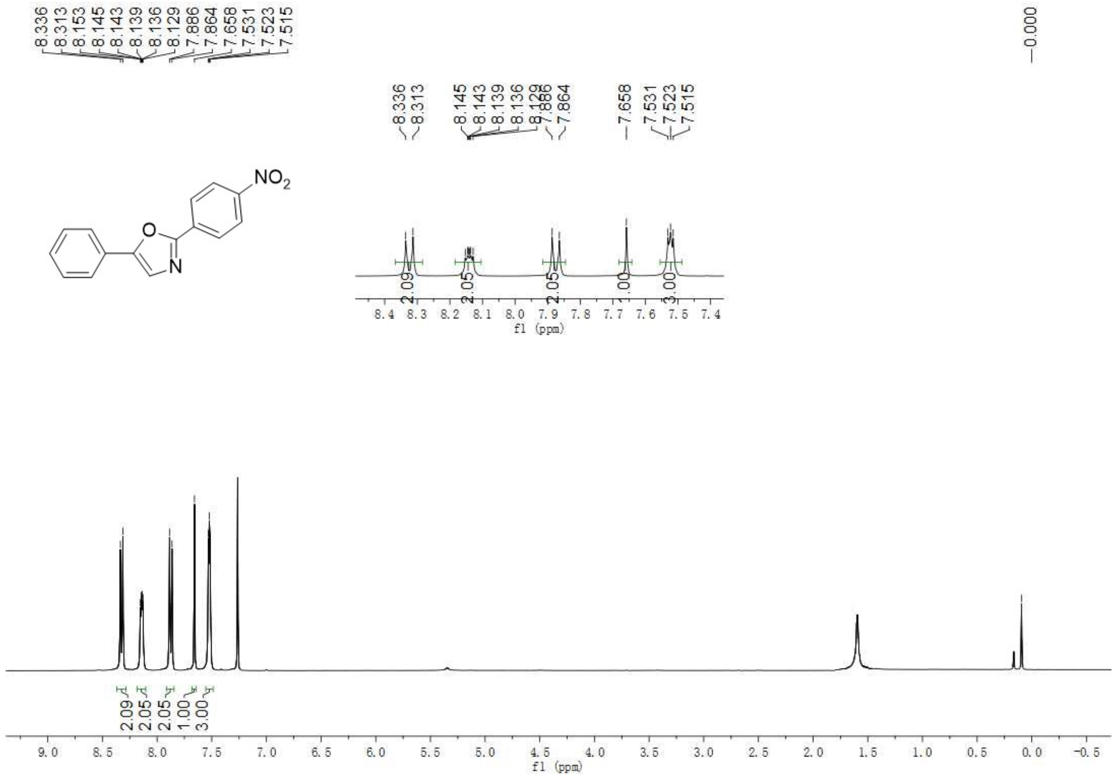
8.336
8.313
8.153
8.145
8.143
8.139
8.136
8.129
7.886
7.864
7.658
7.531
7.523
7.515
0.000
NO2
O
N
2.09
2.05
2.05
1.00
3.00
f1 (ppm)

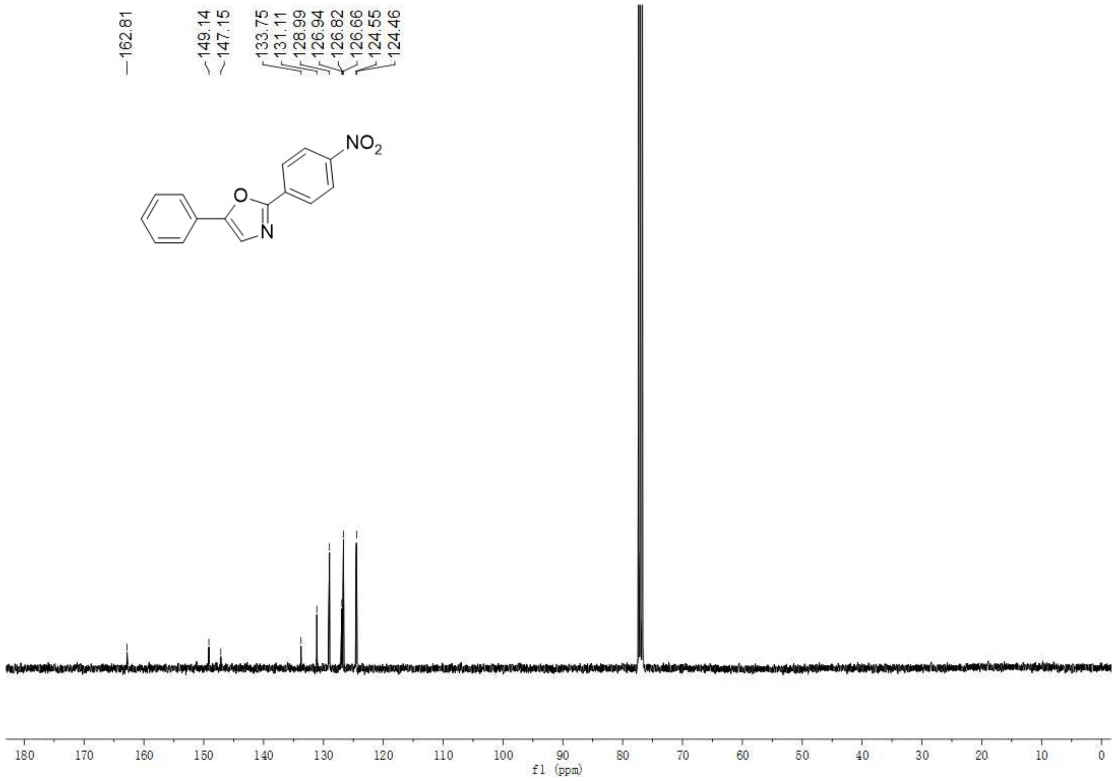
162.81
149.14
147.15
133.75
131.11
128.99
126.94
126.82
126.66
124.55
124.46
NO2
O
N
f1 (ppm)

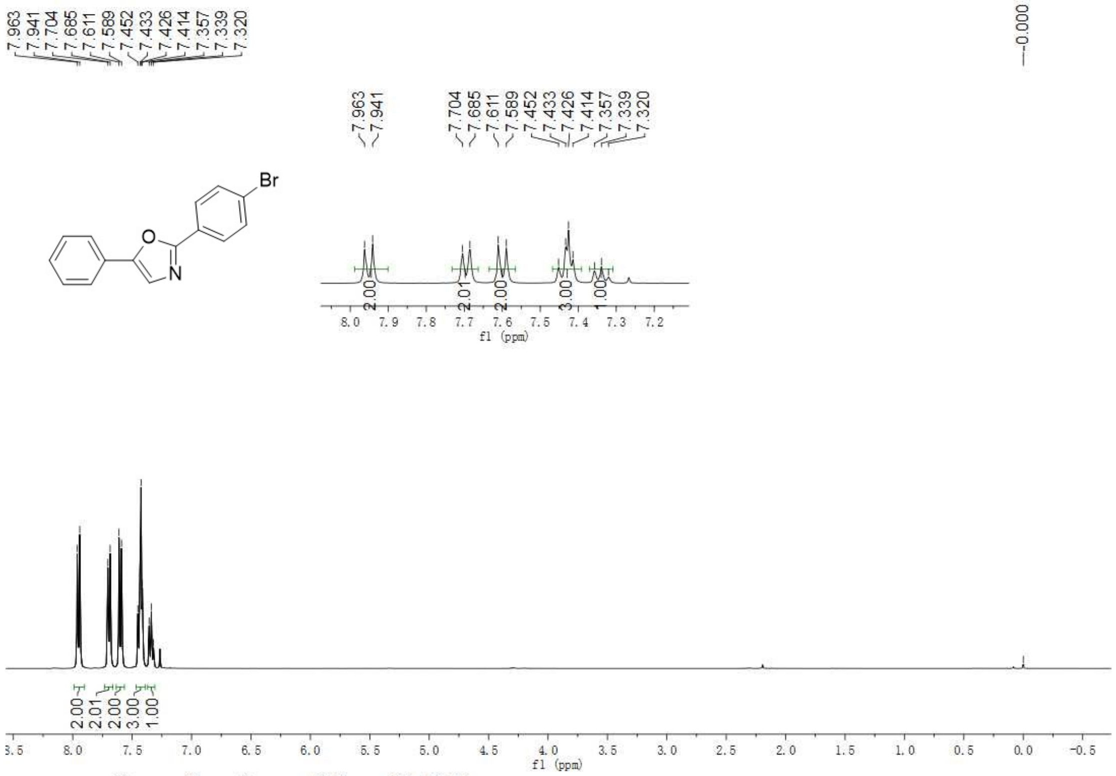
7.963
7.941
7.704
7.685
7.611
7.589
7.452
7.433
7.426
7.414
7.357
7.339
7.320
0.000
Br
O
N
2.00
2.01
2.00
3.00
1.00
f1 (ppm)

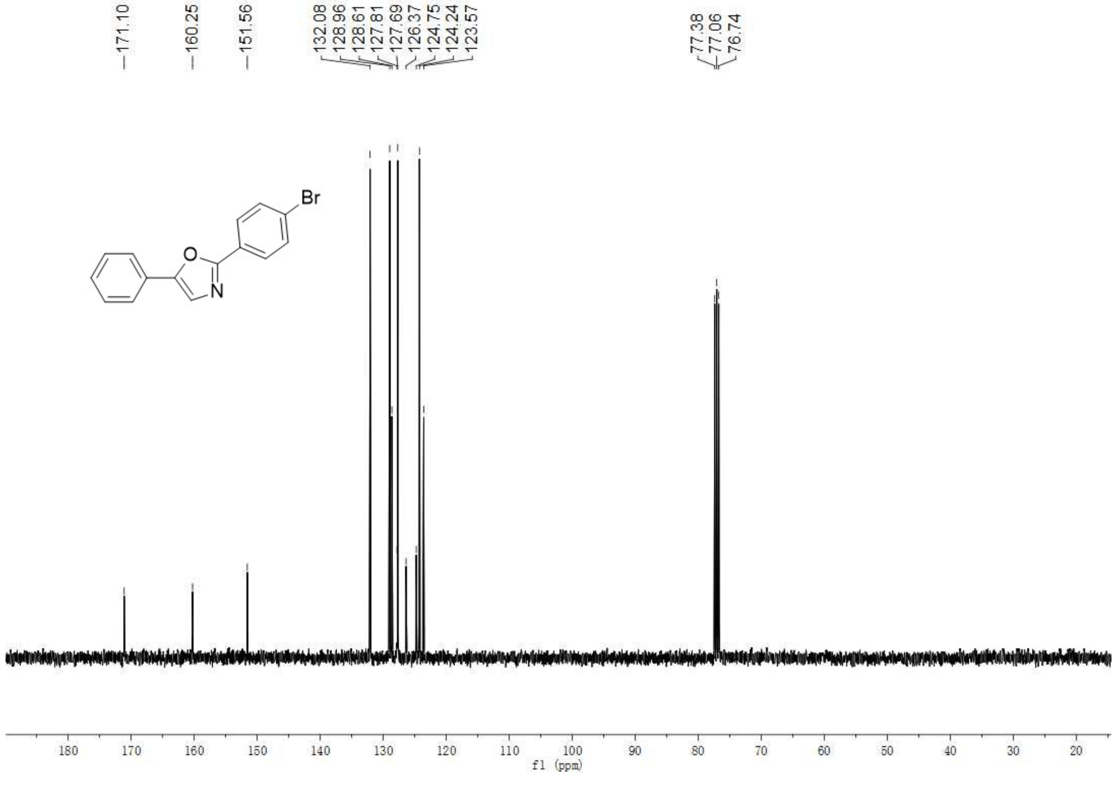
171.10
160.25
151.56
132.08
128.96
128.61
127.81
127.69
126.37
124.75
124.24
123.57
77.38
77.06
76.74
Br
O
N
f1 (ppm)

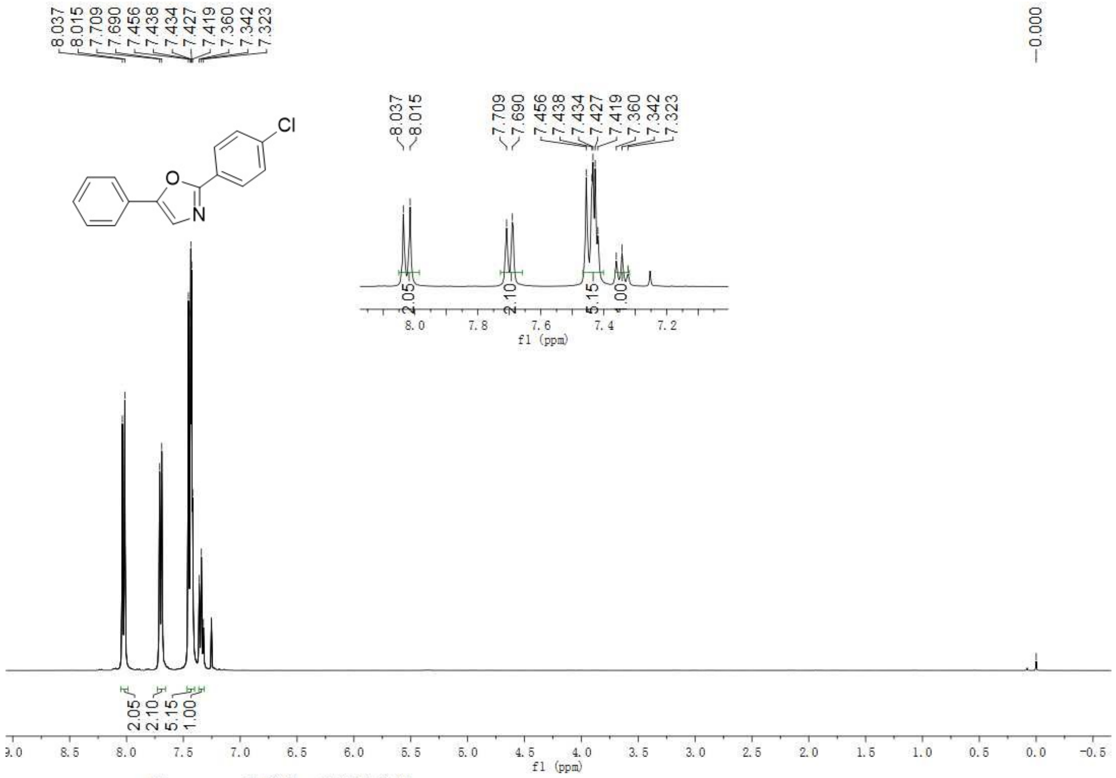

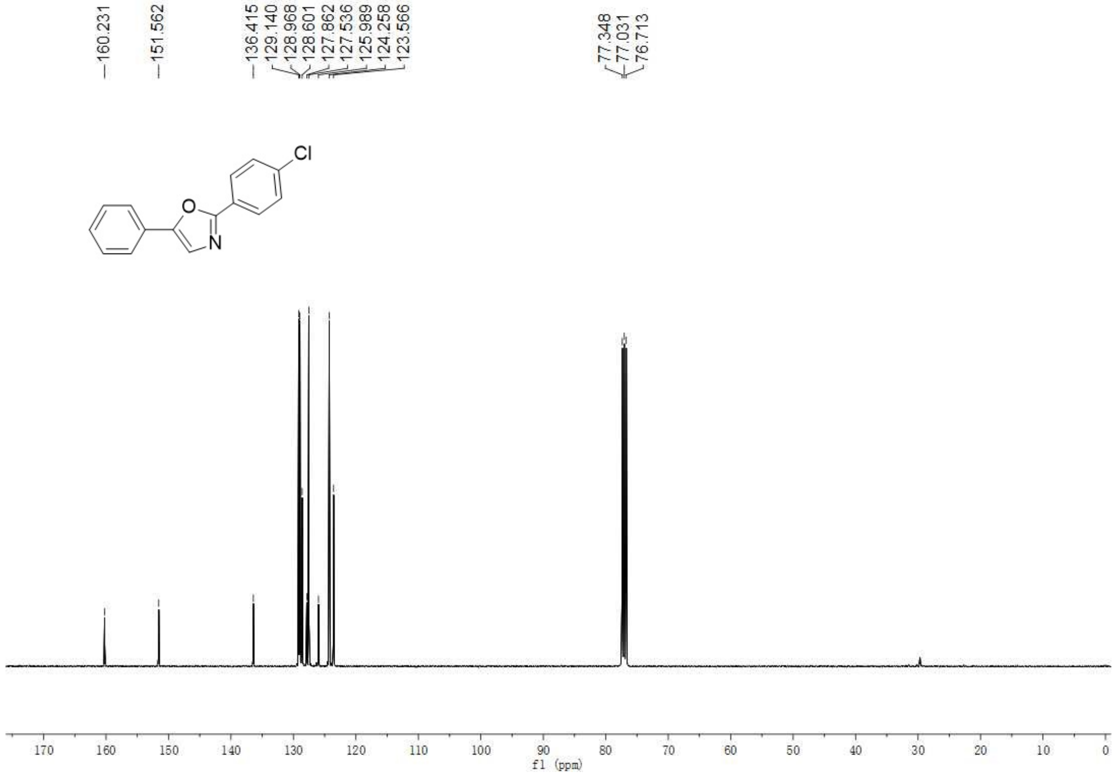

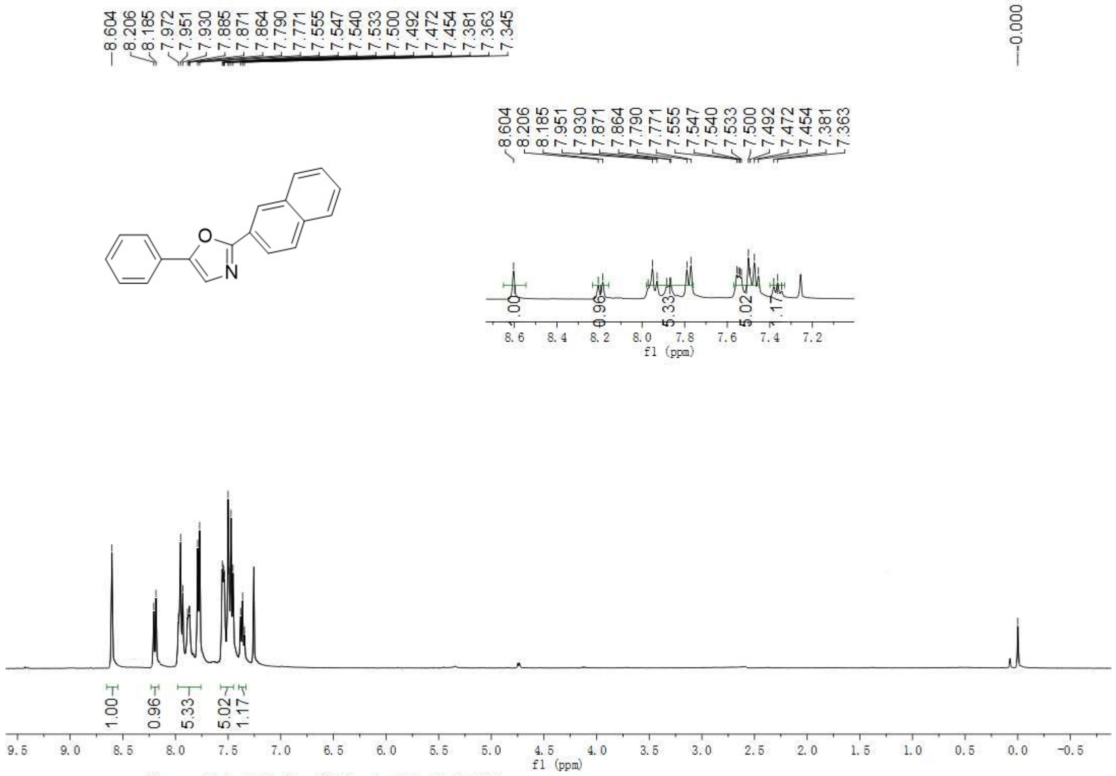

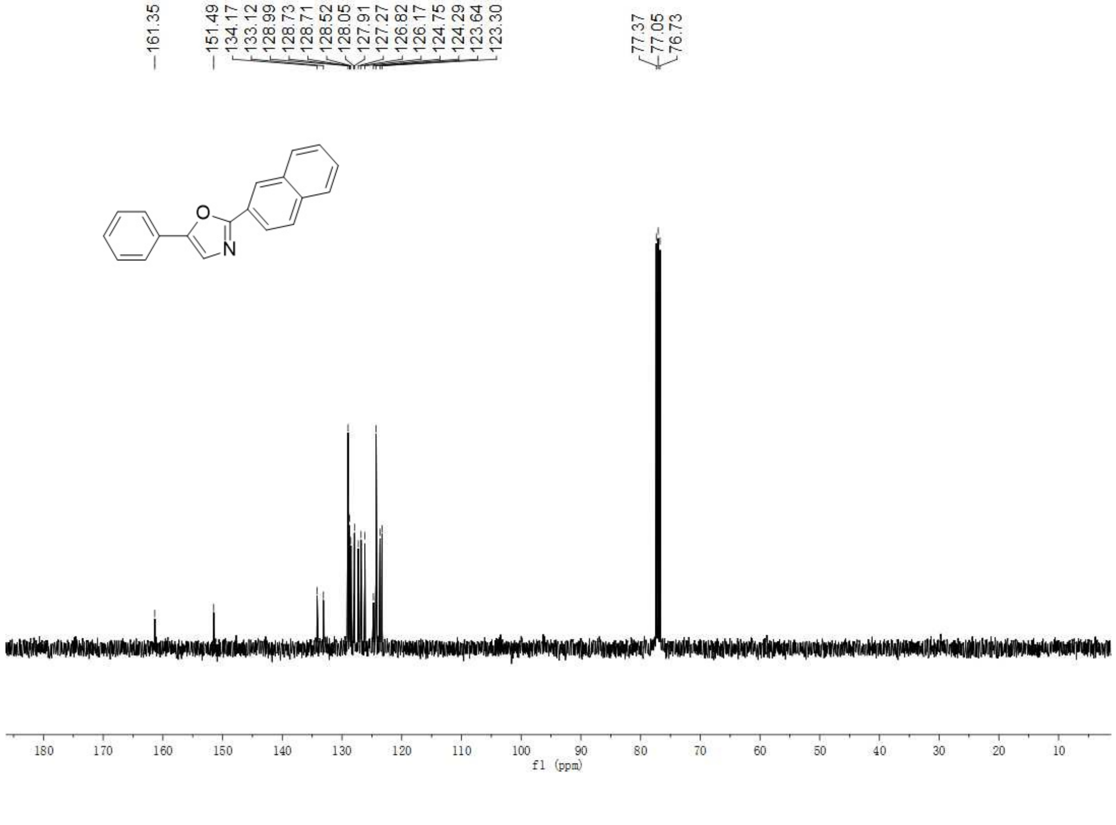

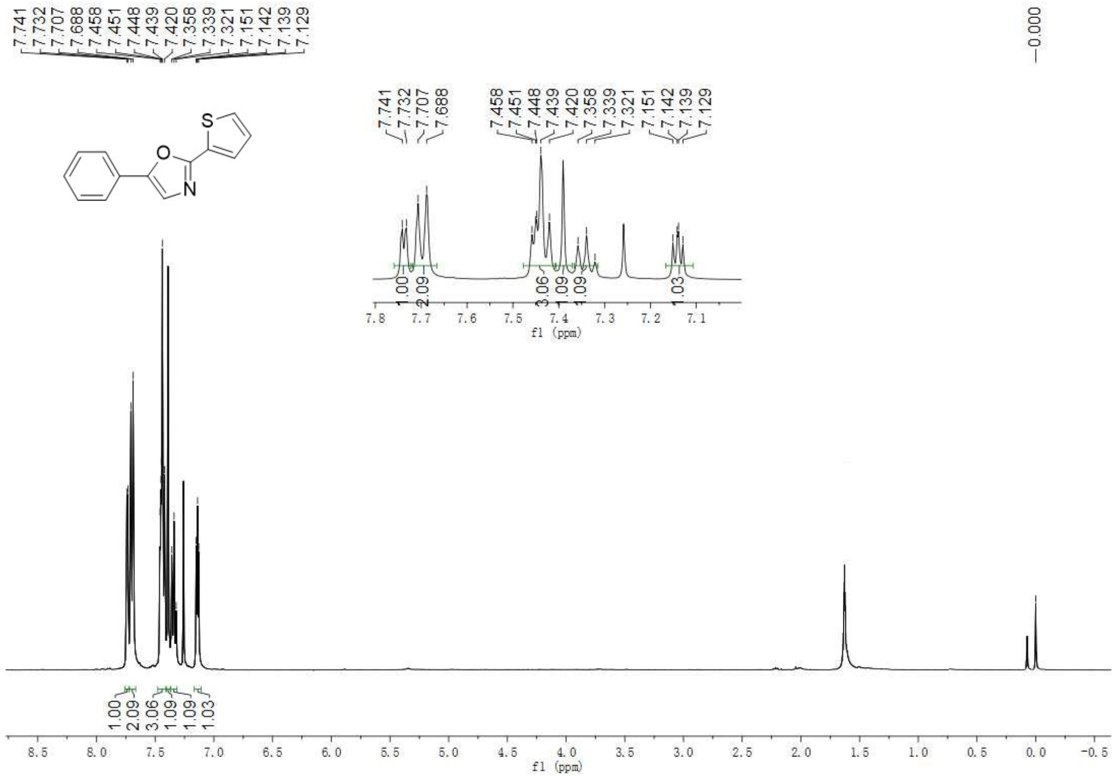
7.741
7.732
7.707
7.688
7.458
7.451
7.448
7.439
7.420
7.358
7.339
7.321
7.151
7.142
7.139
7.129
0.000
S
O
N
1.00
2.09
3.06
1.09
1.09
1.03
f1 (ppm)

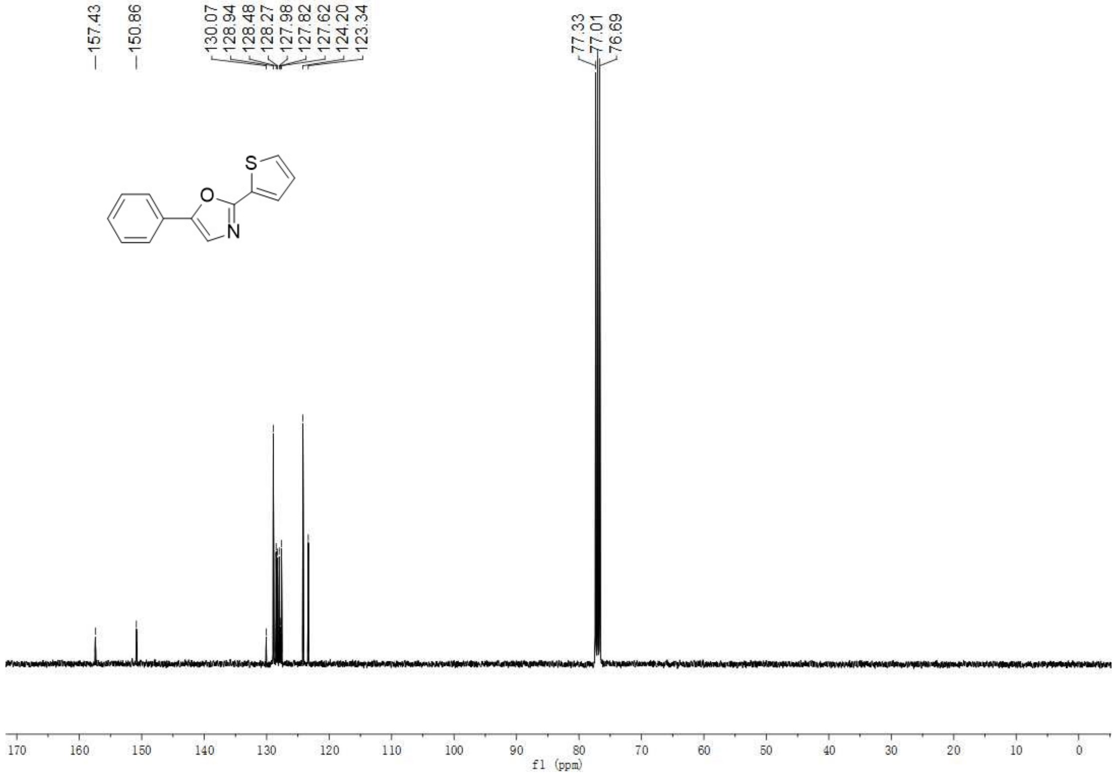
157.43
150.86
130.07
128.94
128.48
128.27
127.98
127.82
127.62
124.20
123.34
77.33
77.01
76.69
S
O
N
f1 (ppm)

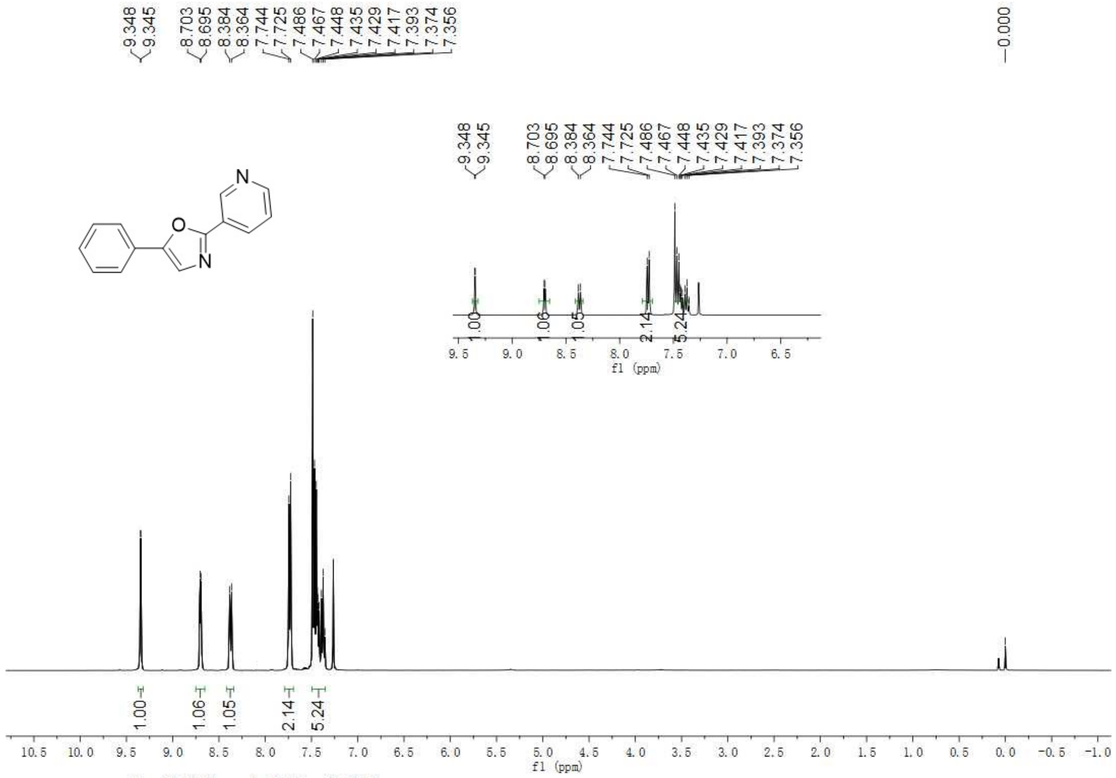
9.348
9.345
8.703
8.695
8.384
8.364
7.744
7.725
7.486
7.467
7.448
7.435
7.429
7.417
7.393
7.374
7.356
0.000
N
O
N
1.00
1.06
1.05
2.14
5.24
f1 (ppm)

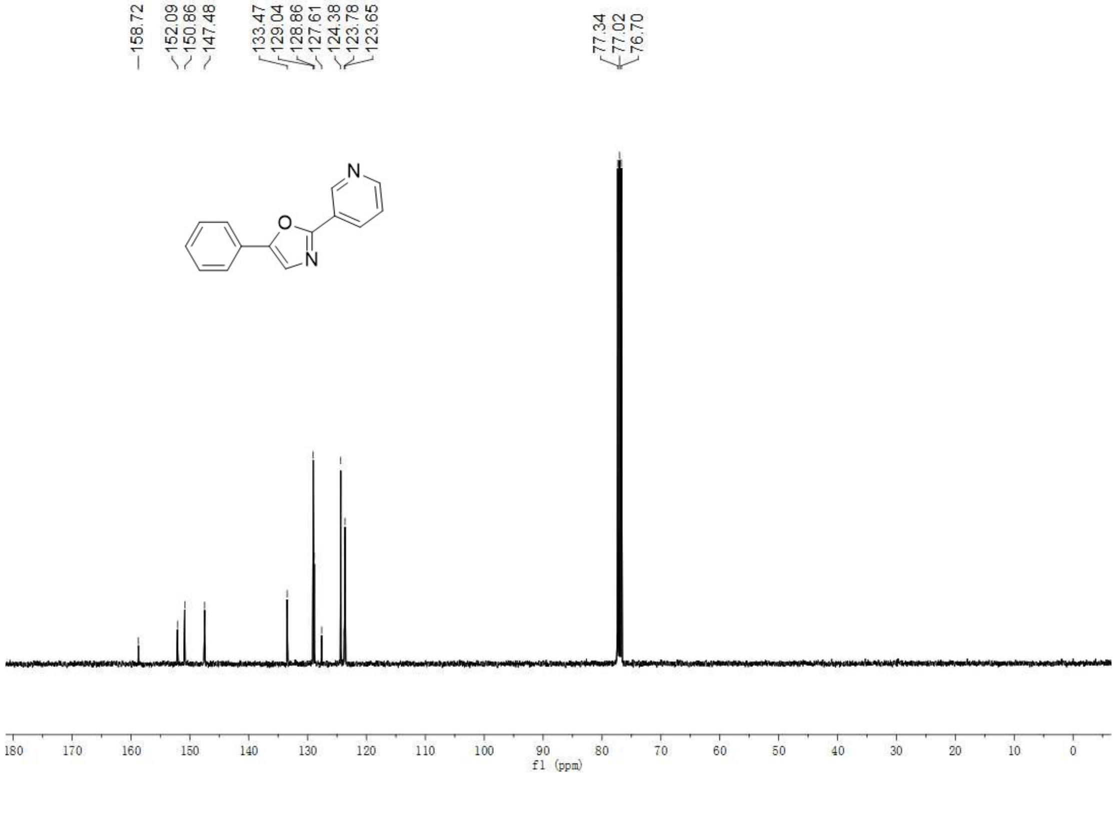
158.72
152.09
150.86
147.48
133.47
129.04
128.86
127.61
124.38
123.78
123.65
77.34
77.02
76.70
N
O
N
f1 (ppm)

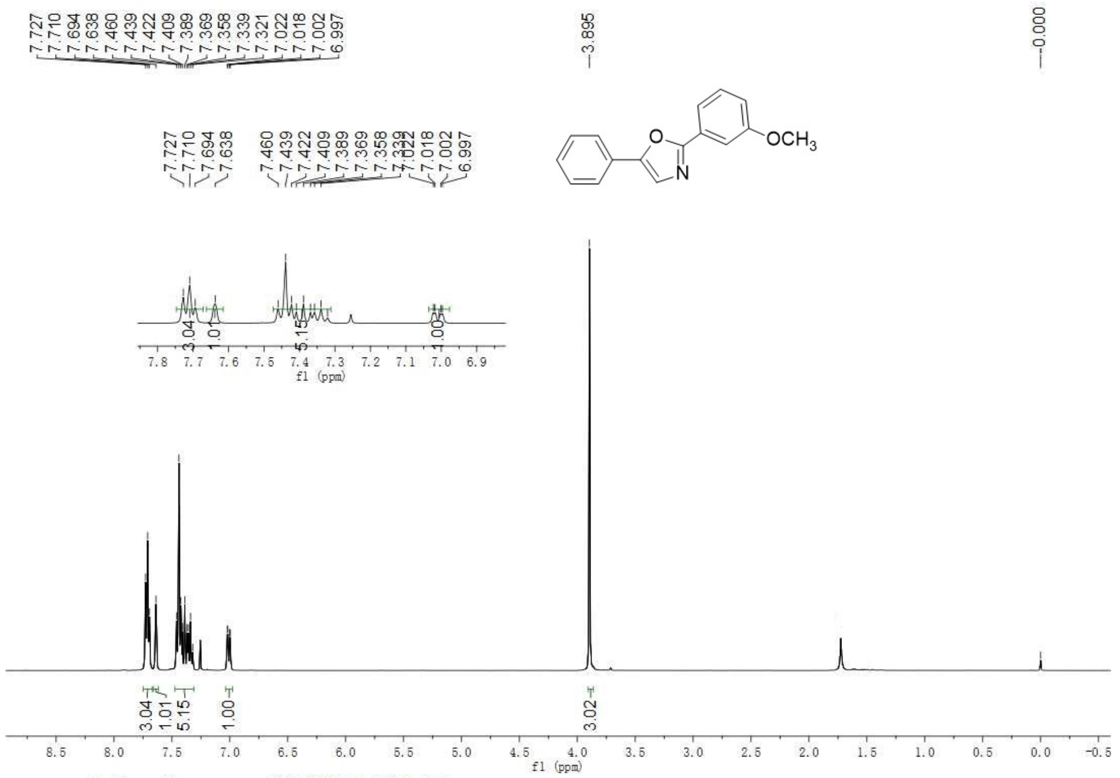

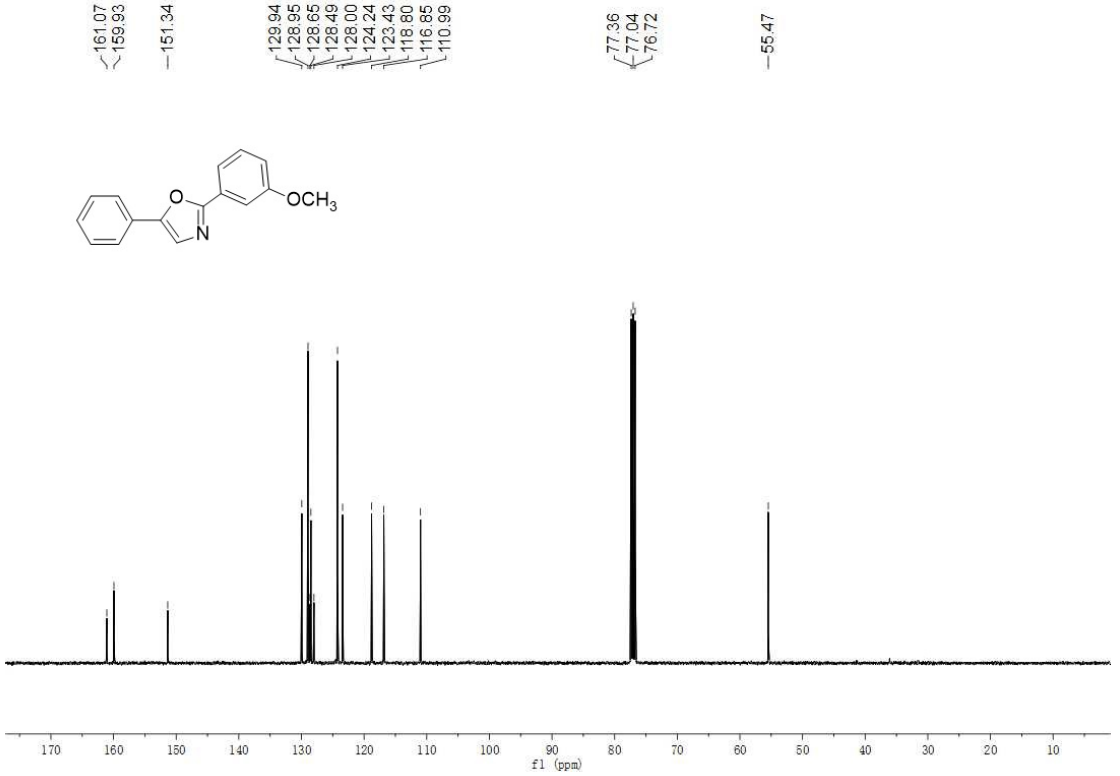

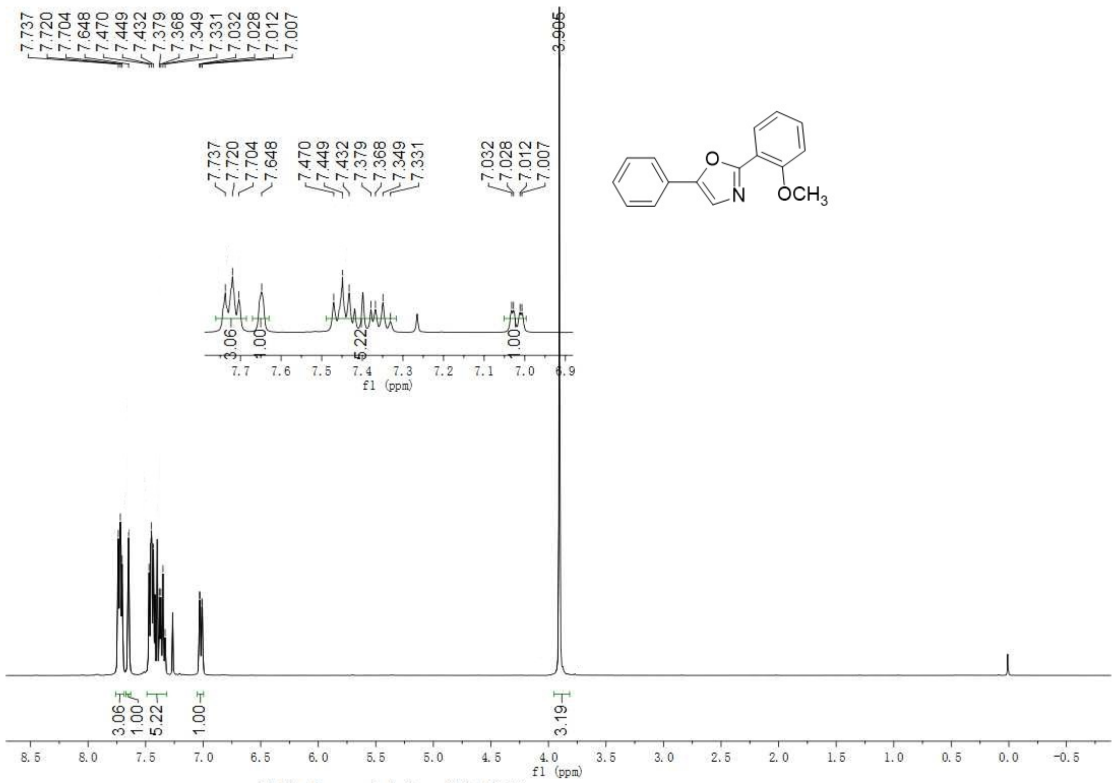
7.737 7.720 7.704 7.648 7.470 7.449 7.432 7.379 7.368 7.349 7.331 7.032 7.028 7.012 7.007
3.905
3.06
1.00
5.22
1.00
3.19
OCH3
O
N
f1 (ppm)

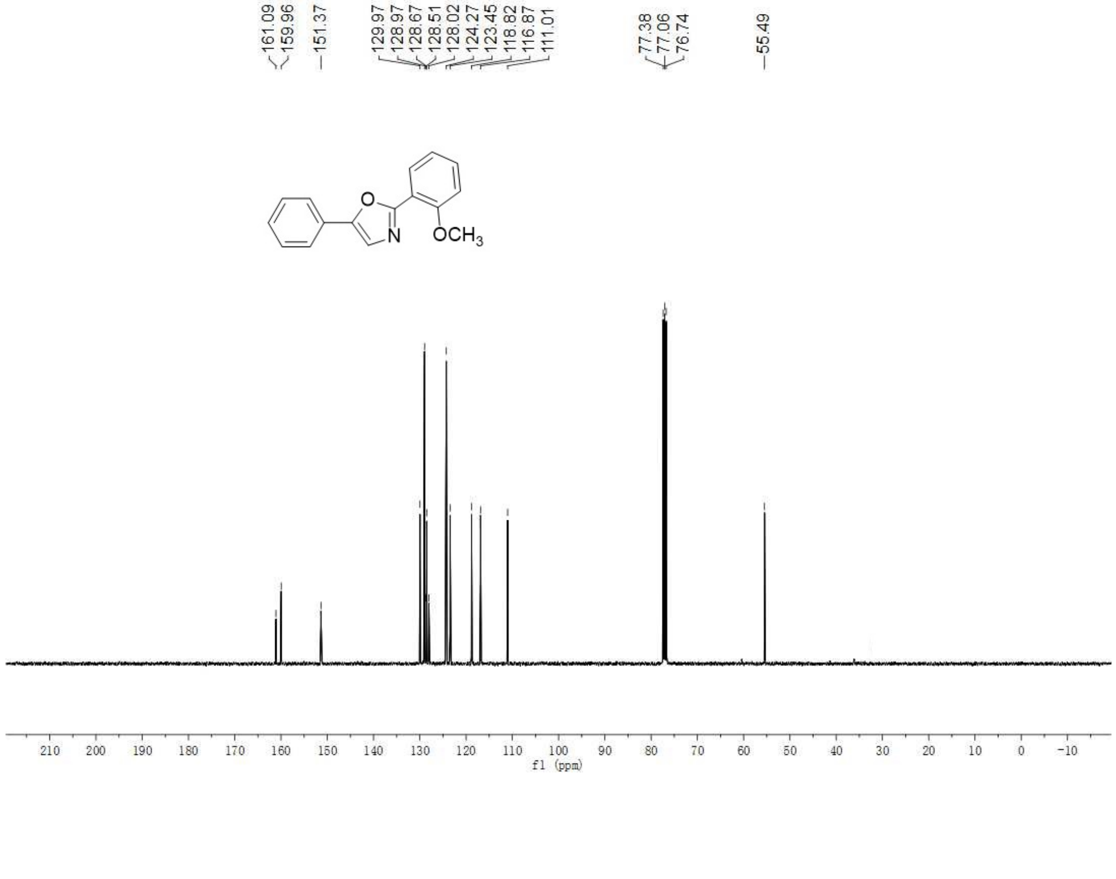
161.09 159.96 151.37 129.97 128.97 128.67 128.51 128.02 124.27 123.45 118.82 116.87 111.01 77.38 77.06 76.74 55.49
OCH3
O
N
f1 (ppm)

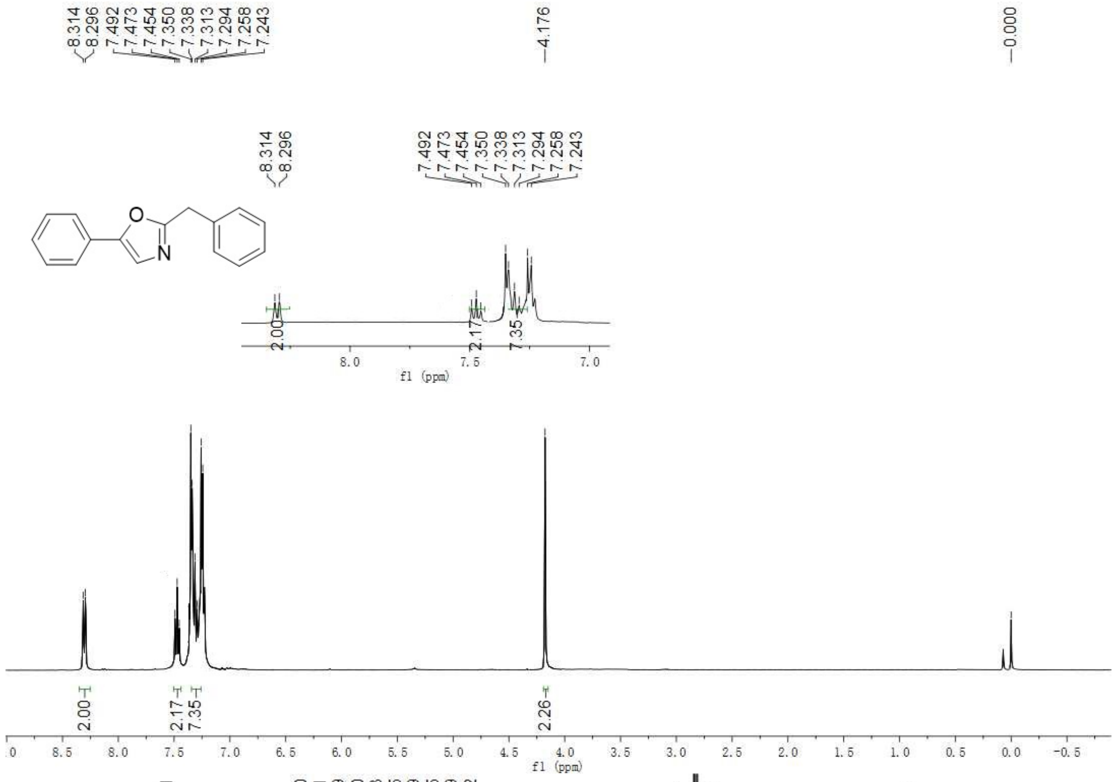

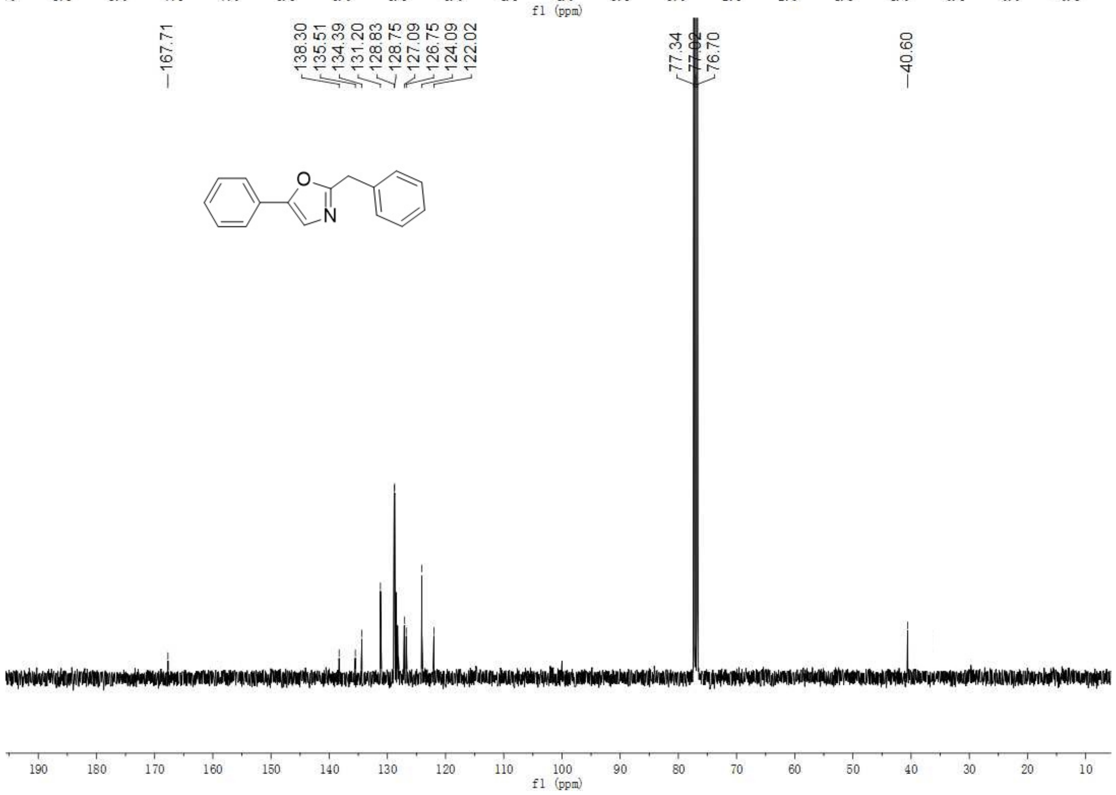

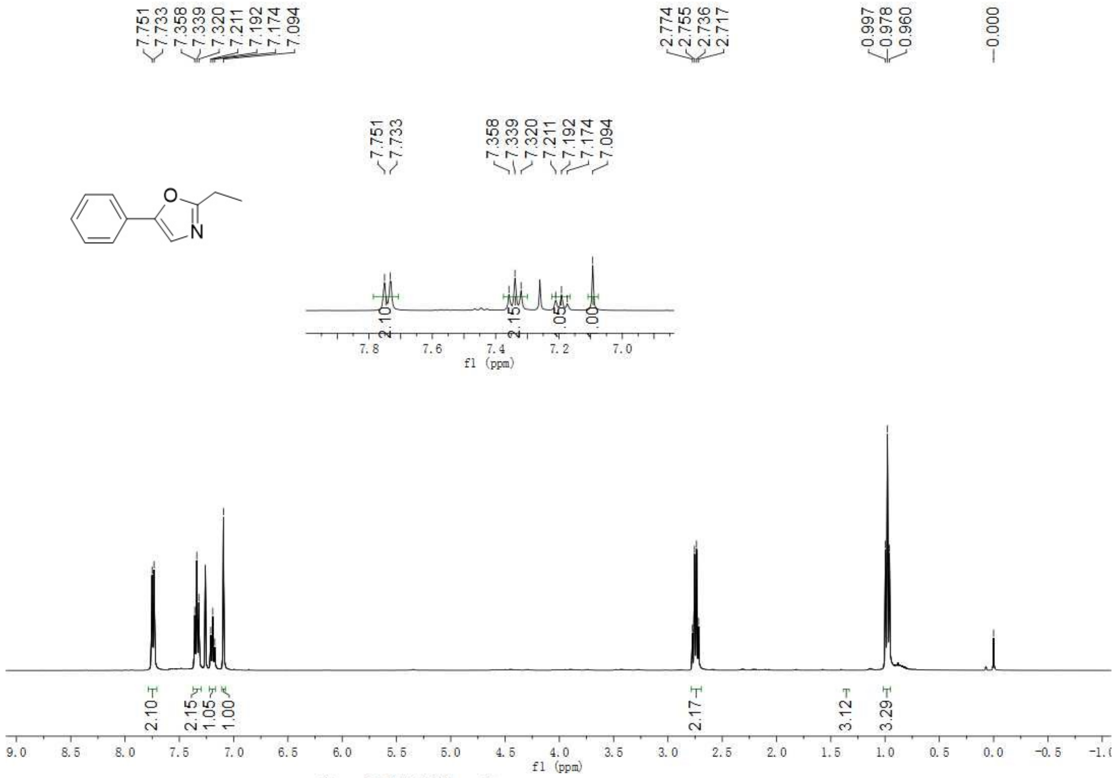
7.751
7.733
7.358
7.339
7.320
7.211
7.192
7.174
7.094
2.774
2.755
2.736
2.717
0.997
0.978
0.960
0.000
O
N
2.10
2.15
1.05
1.00
f1 (ppm)
2.17
3.12
3.29
f1 (ppm)

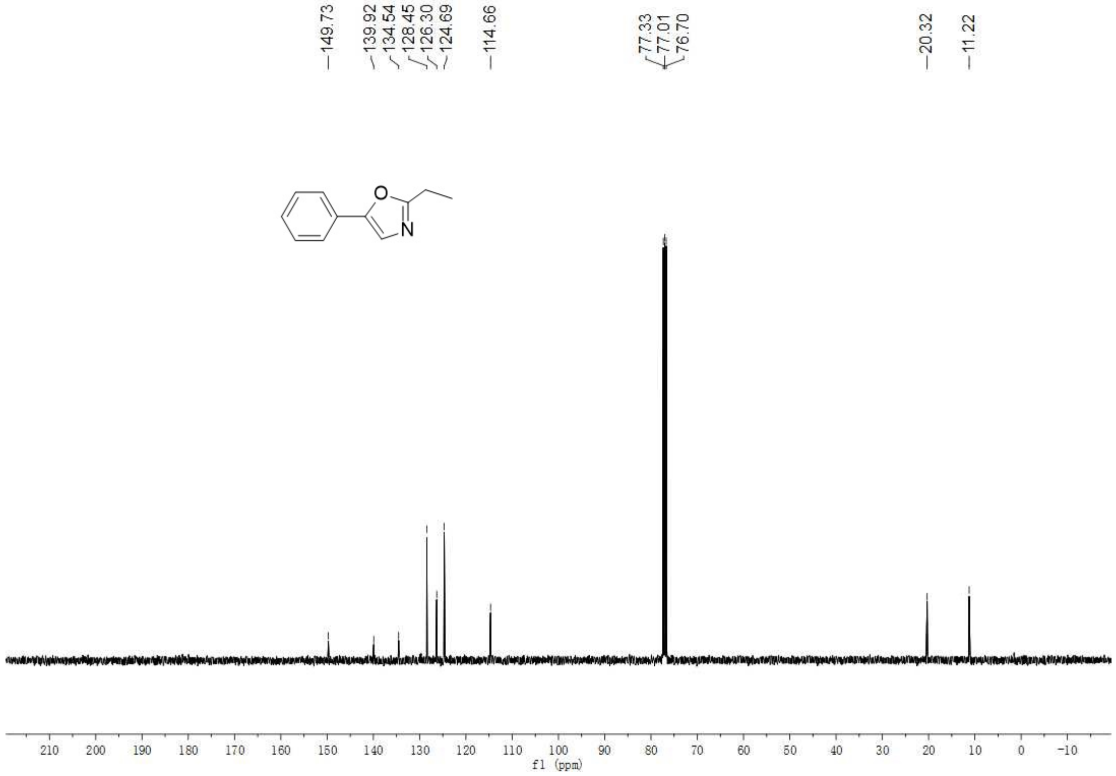
149.73
139.92
134.54
128.45
126.30
124.69
114.66
77.33
77.01
76.70
20.32
11.22
O
N
f1 (ppm)

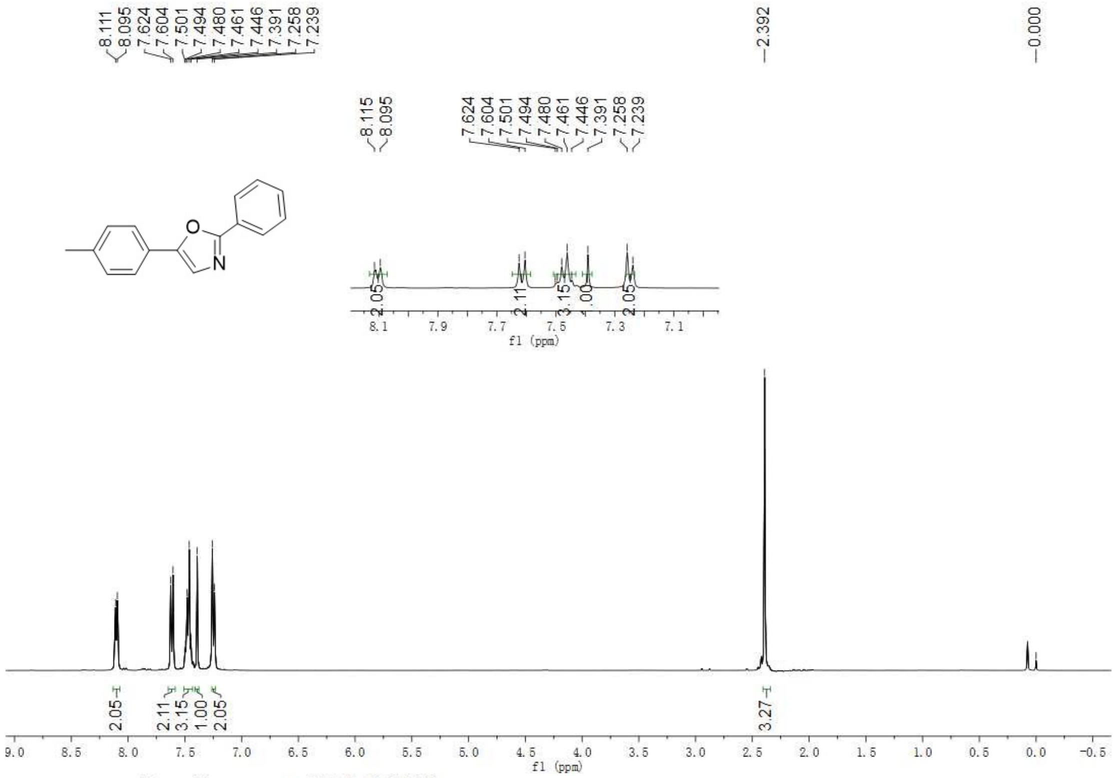
8.111
8.095
7.624
7.604
7.501
7.494
7.480
7.461
7.446
7.391
7.258
7.239
2.392
0.000
8.115
8.095
N
O
2.05
2.11
3.15
1.00
2.05
3.27
f1 (ppm)

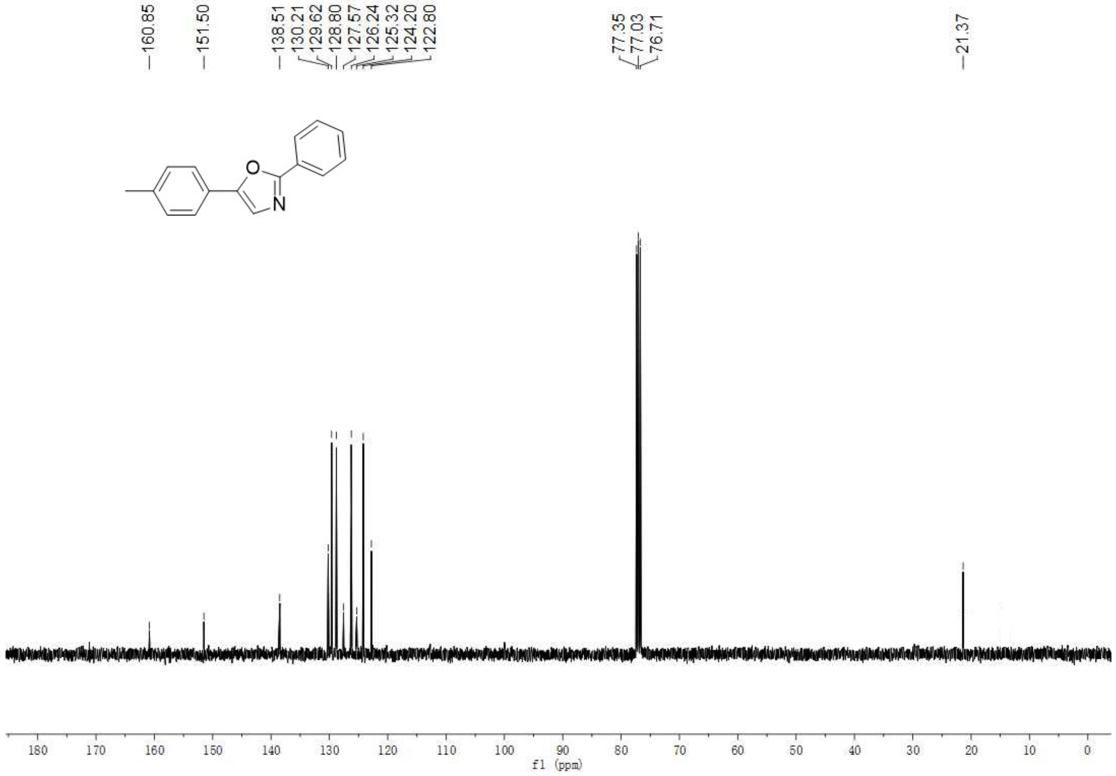
160.85
151.50
138.51
130.21
129.62
128.80
127.57
126.24
125.32
124.20
122.80
77.35
77.03
76.71
21.37
N
O
f1 (ppm)

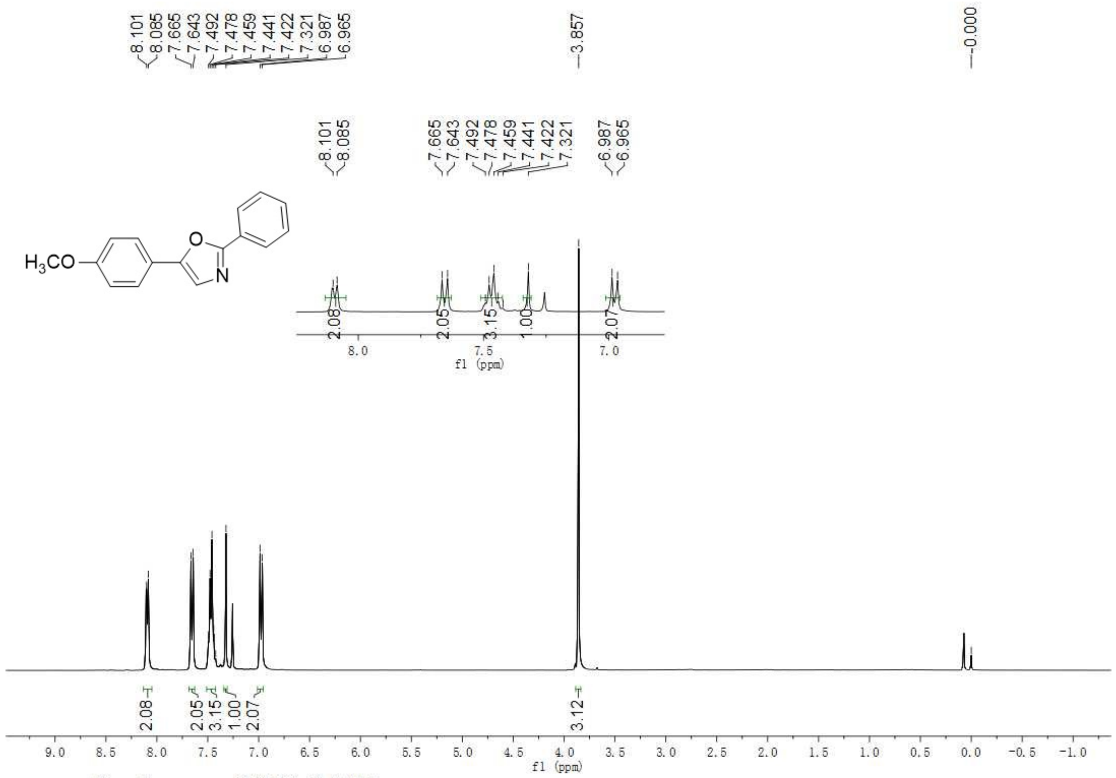
8.101
8.085
7.665
7.643
7.492
7.478
7.459
7.441
7.422
7.321
6.987
6.965
3.857
0.000
H_3CO
O
N
2.08
2.05
3.15
1.00
2.07
8.0
7.5
7.0
fl (ppm)
3.12
9.0 8.5 8.0 7.5 7.0 6.5 6.0 5.5 5.0 4.5 4.0 3.5 3.0 2.5 2.0 1.5 1.0 0.5 0.0 -0.5 -1.0
fl (ppm)

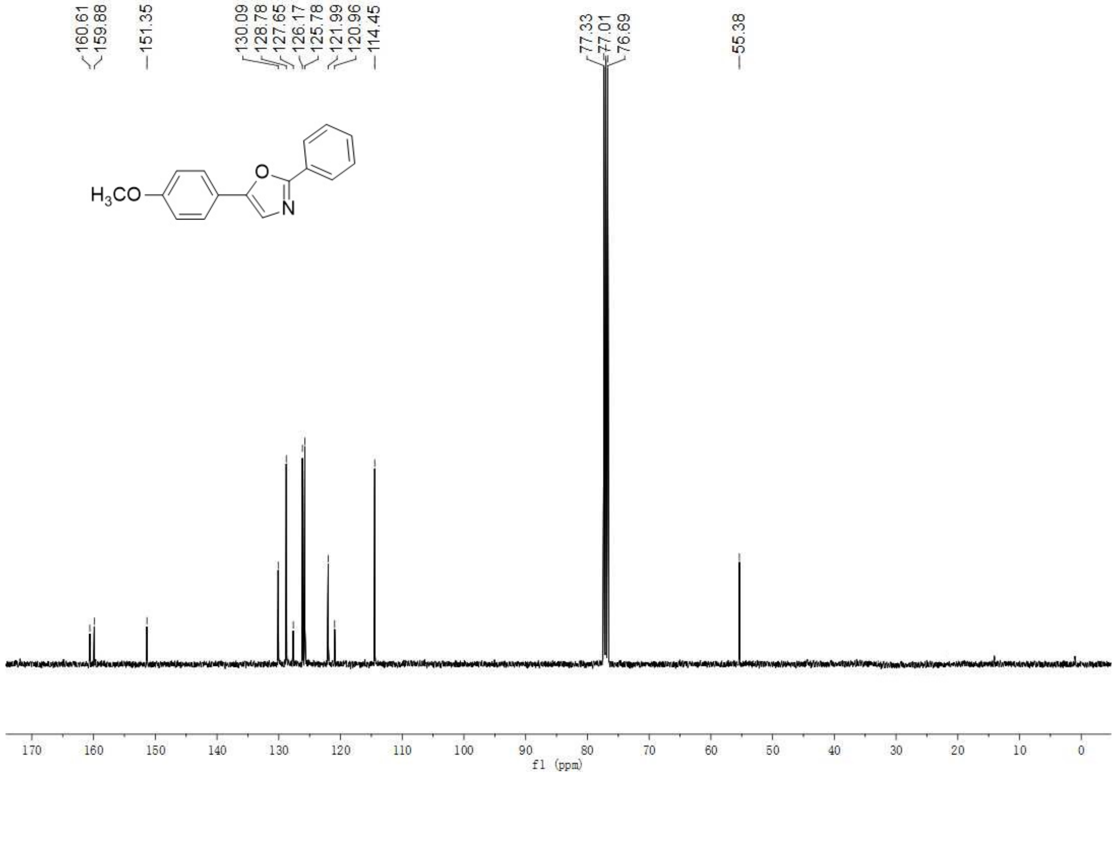
160.61
159.88
151.35
130.09
128.78
127.65
126.17
125.78
121.99
120.96
114.45
77.33
77.01
76.69
55.38
H_3CO
O
N
170 160 150 140 130 120 110 100 90 80 70 60 50 40 30 20 10 0
fl (ppm)

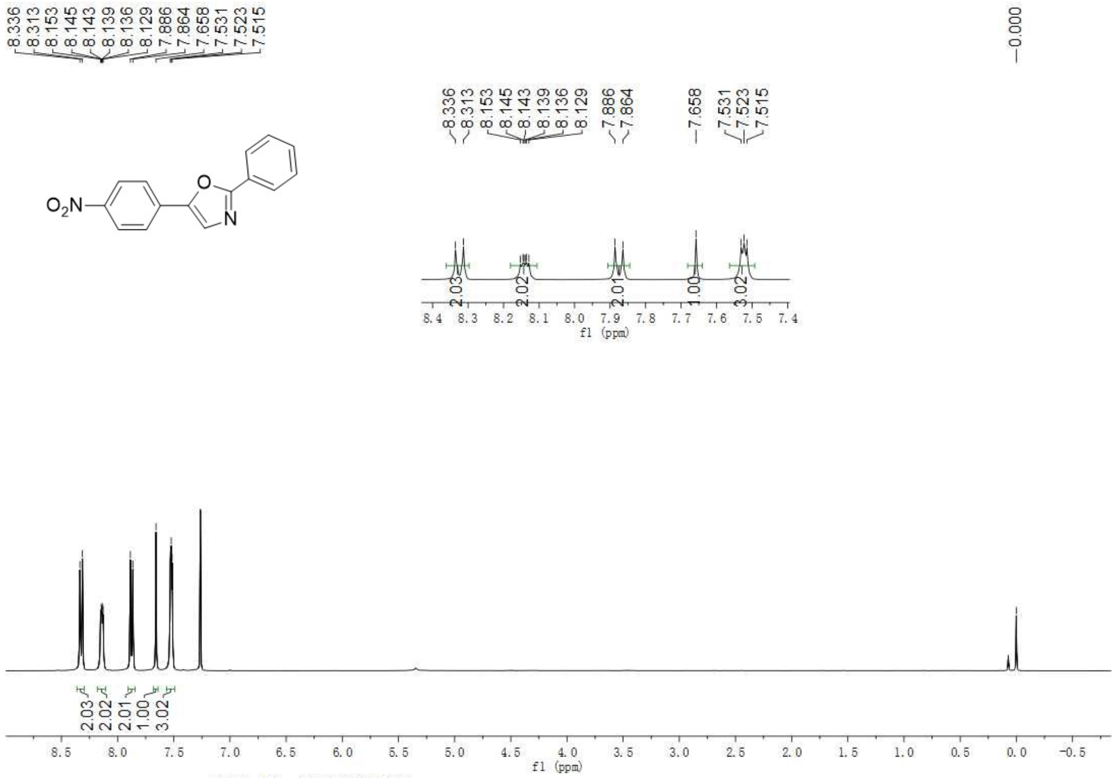
8.336
8.313
8.153
8.145
8.143
8.139
8.136
8.129
7.886
7.864
7.658
7.531
7.523
7.515
0.000
O_2N
O
N
2.03
2.02
2.01
1.00
3.02
fl (ppm)

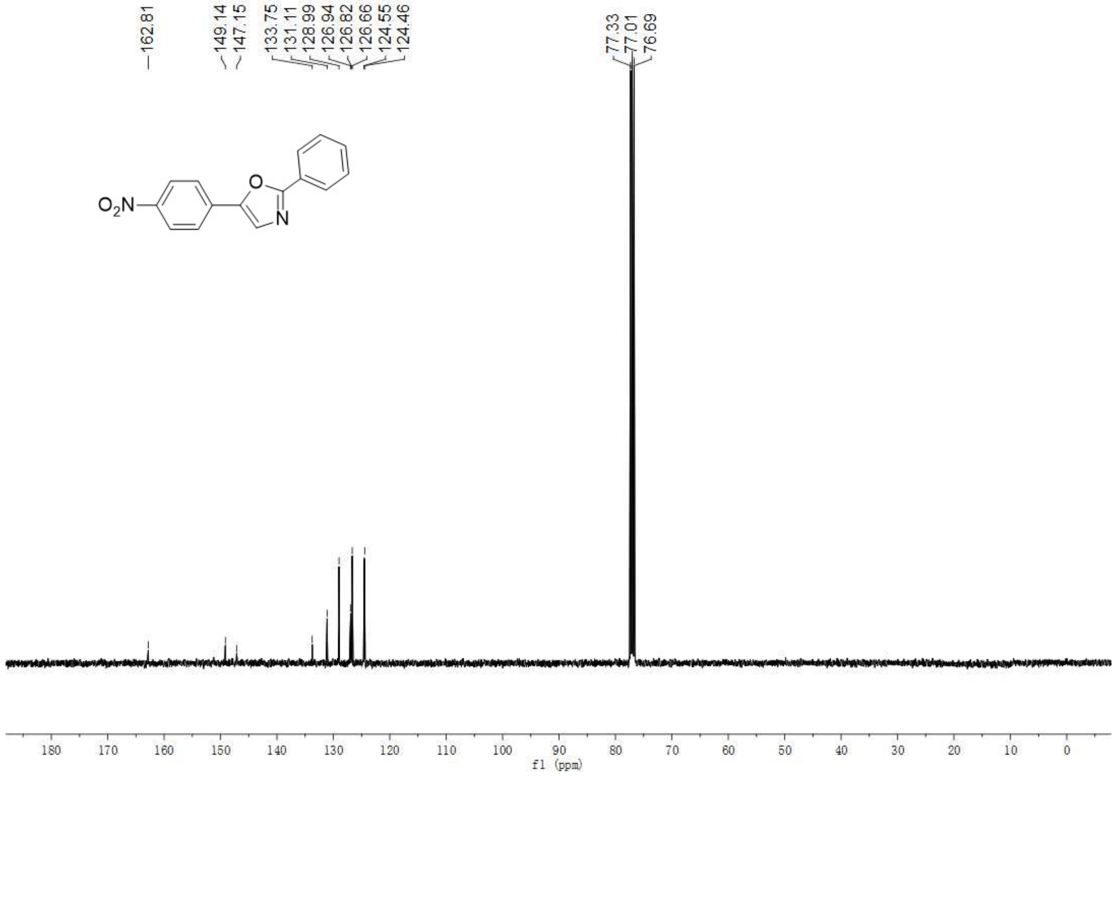
162.81
149.14
147.15
133.75
131.11
128.99
126.94
126.82
126.66
124.55
124.46
77.33
77.01
76.69
O_2N
O
N
fl (ppm)

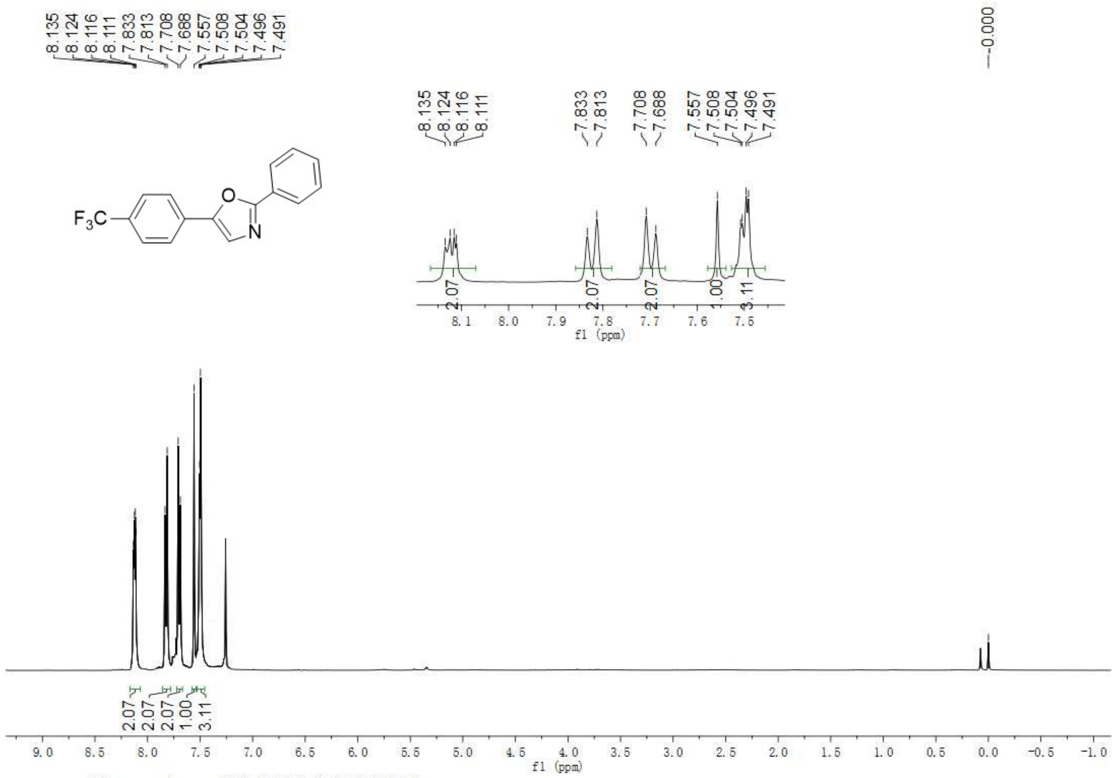
8.135
8.124
8.116
8.111
7.833
7.813
7.708
7.688
7.557
7.508
7.504
7.496
7.491
0.000
F_3C
O
N
2.07
2.07
2.07
1.00
3.11
f1 (ppm)

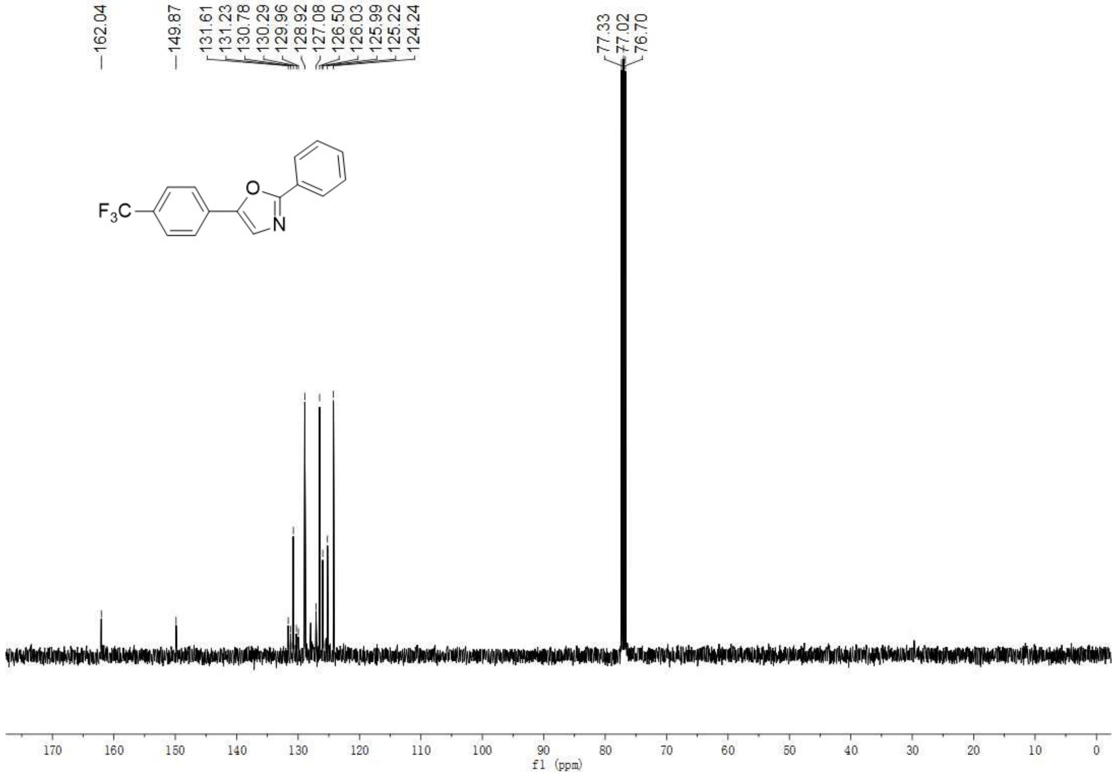
162.04
149.87
131.61
131.23
130.78
130.29
129.96
128.92
127.08
126.50
126.03
125.99
125.22
124.24
77.33
77.02
76.70
F_3C
O
N
f1 (ppm)

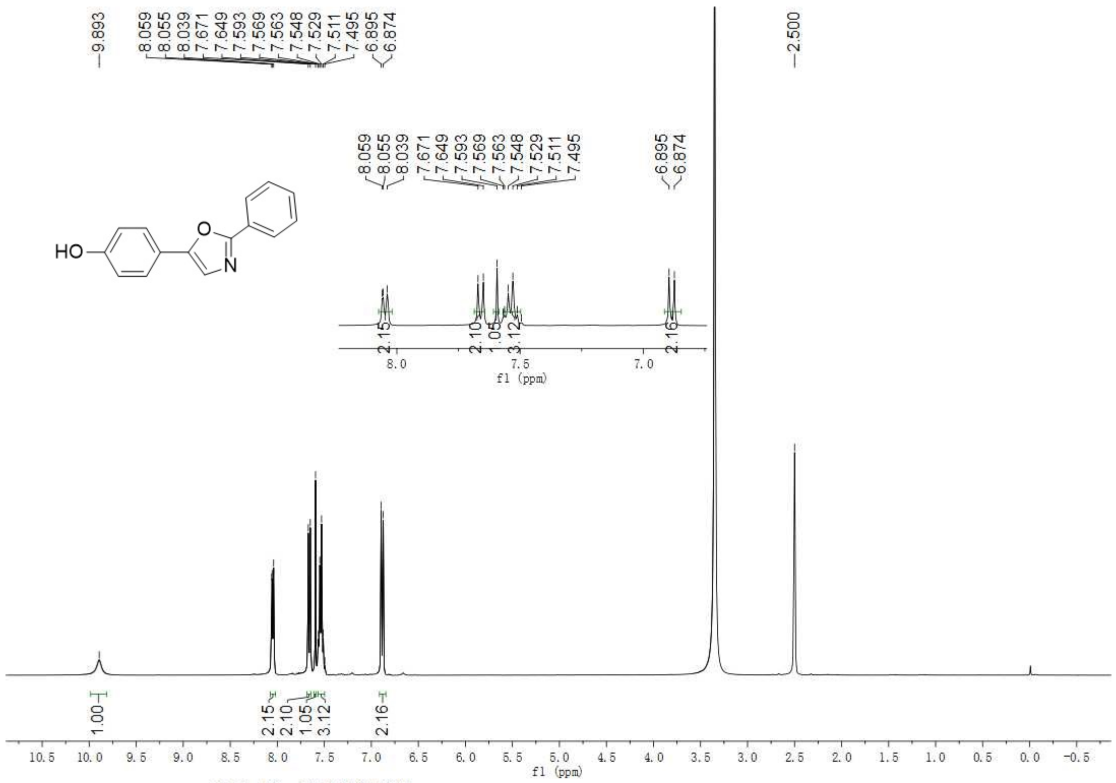
9.893
8.059
8.055
8.039
7.671
7.649
7.593
7.569
7.563
7.548
7.529
7.511
7.495
6.895
6.874
2.500
HO
O
N
2.15
2.10
1.05
3.12
2.16
1.00
f1 (ppm)

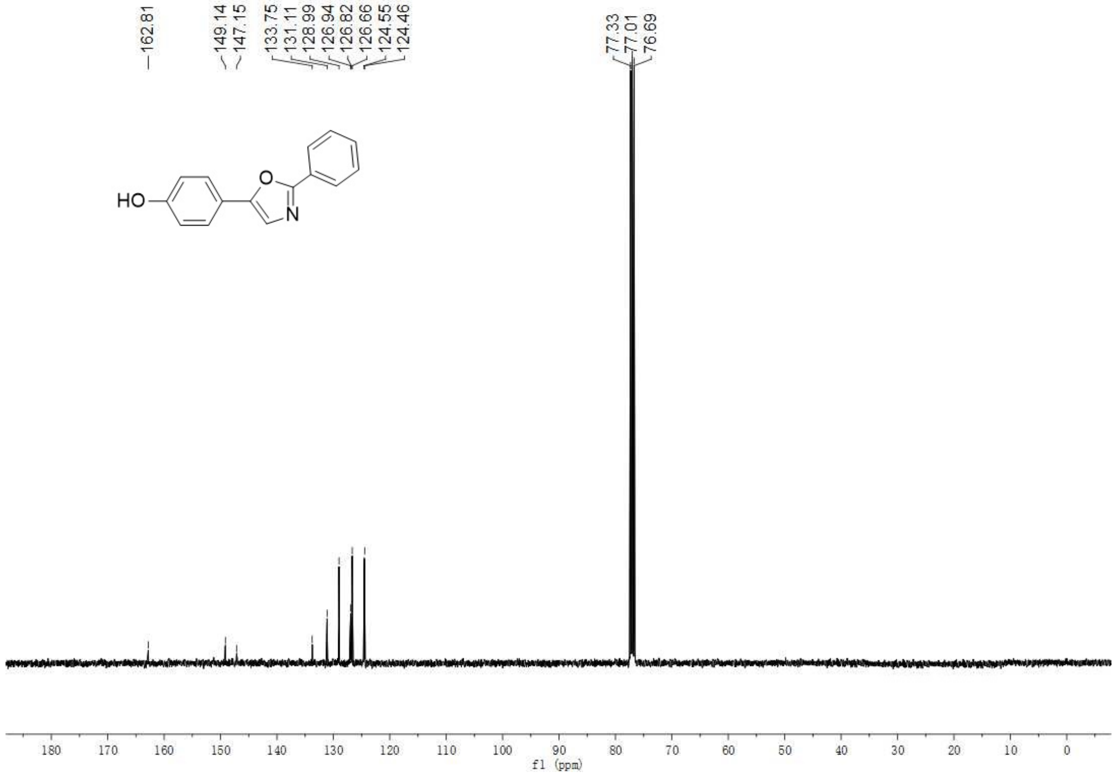
162.81
149.14
147.15
133.75
131.11
128.99
126.94
126.82
126.66
124.55
124.46
77.33
77.01
76.69
HO
O
N
f1 (ppm)

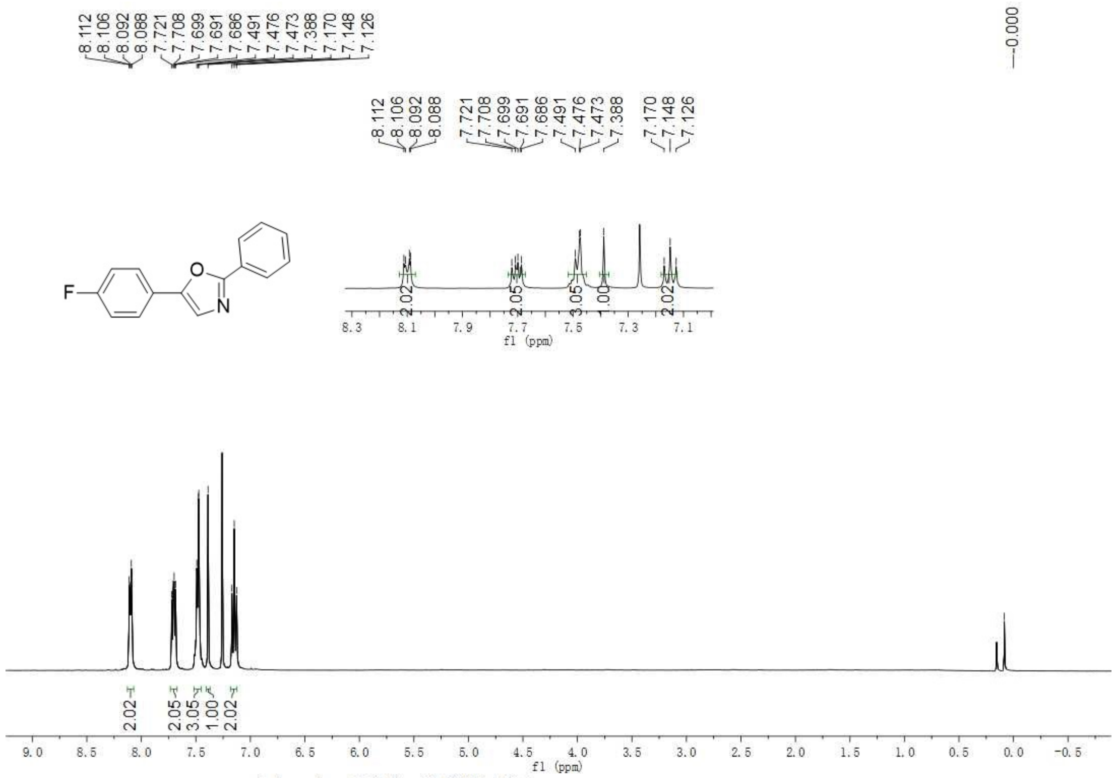
8.112
8.106
8.092
8.088
7.721
7.708
7.699
7.691
7.686
7.491
7.476
7.473
7.388
7.170
7.148
7.126
0.000
F
O
N
2.02
2.05
3.05
1.00
2.02
f1 (ppm)

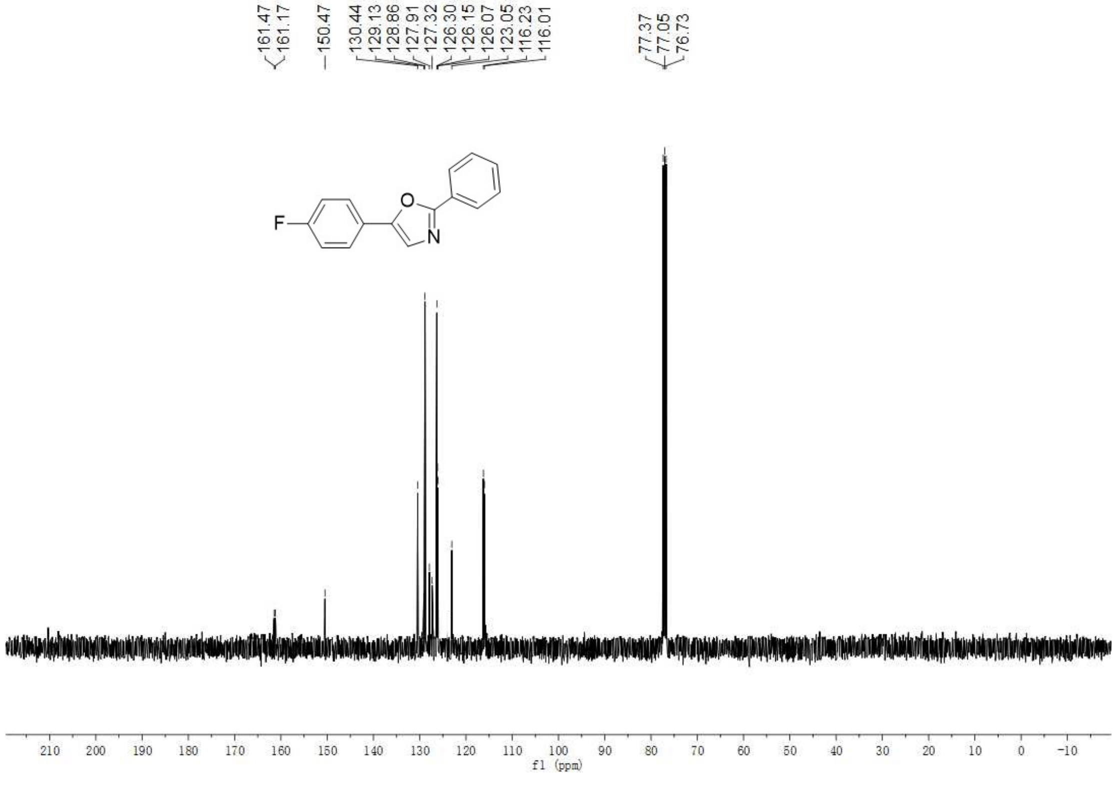
161.47
161.17
150.47
130.44
129.13
128.86
127.91
127.32
126.30
126.15
126.07
123.05
116.23
116.01
77.37
77.05
76.73
F
O
N
f1 (ppm)

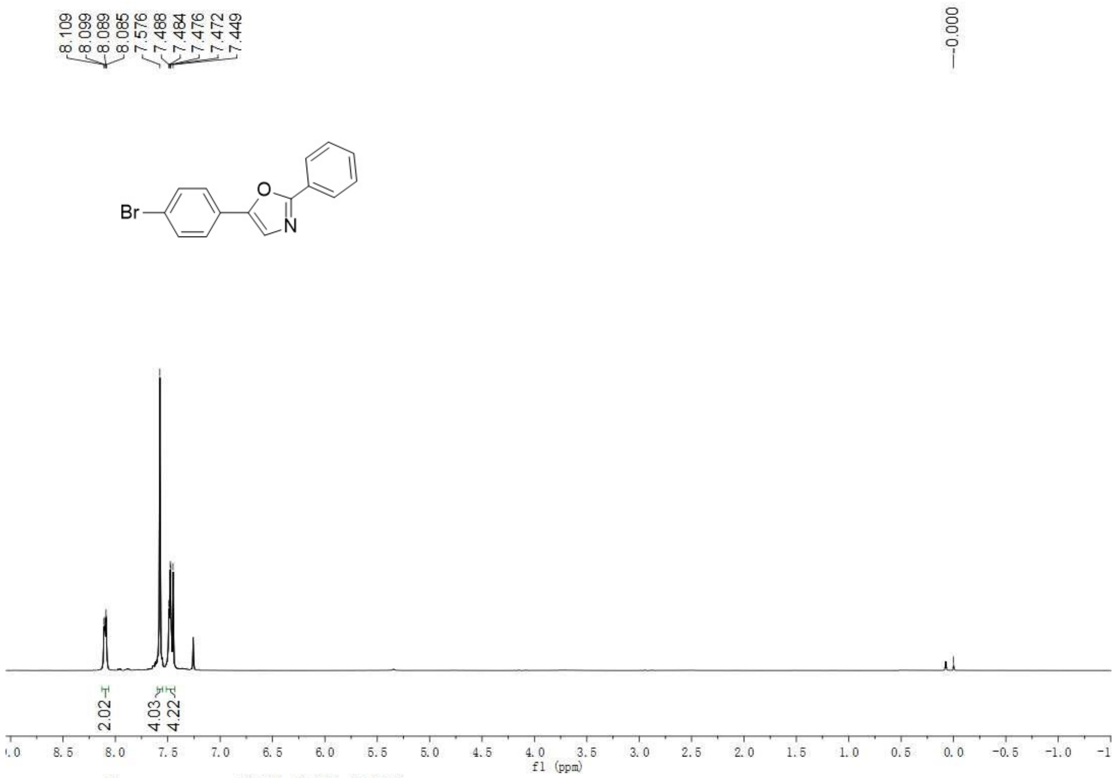
8.109
8.099
8.089
8.085
7.576
7.488
7.484
7.476
7.472
7.449
0.000
Br
O
N
2.02
4.03
4.22
f1 (ppm)

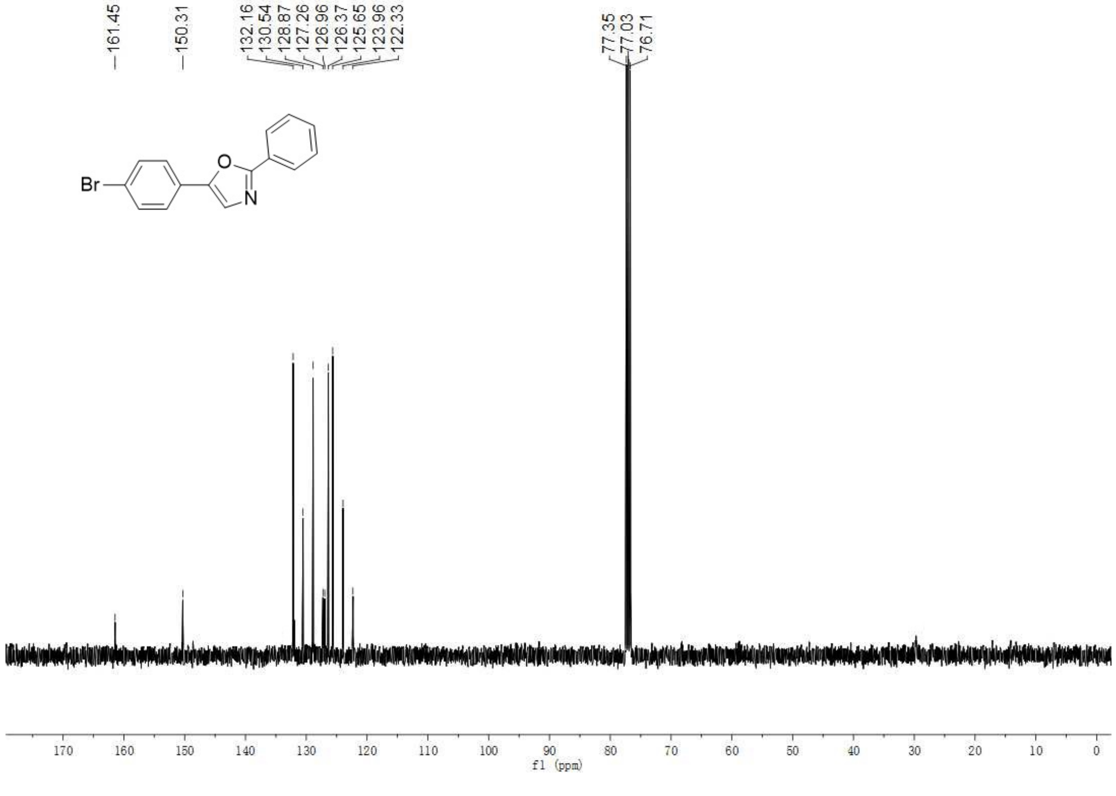
161.45
150.31
132.16
130.54
128.87
127.26
126.96
126.37
125.65
123.96
122.33
77.35
77.03
76.71
Br
O
N
f1 (ppm)

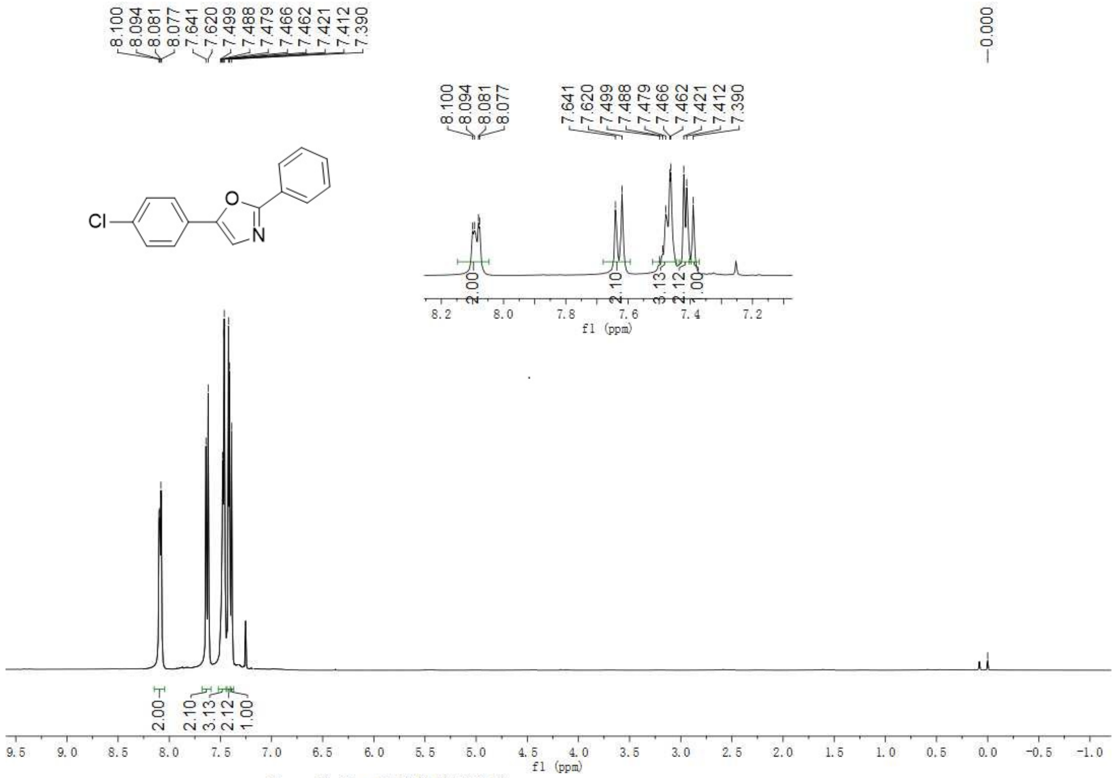
8.100
8.094
8.081
8.077
7.641
7.620
7.499
7.488
7.479
7.466
7.462
7.421
7.412
7.390
0.000
Cl
O
N
2.00
2.10
3.13
2.12
1.00
f1 (ppm)

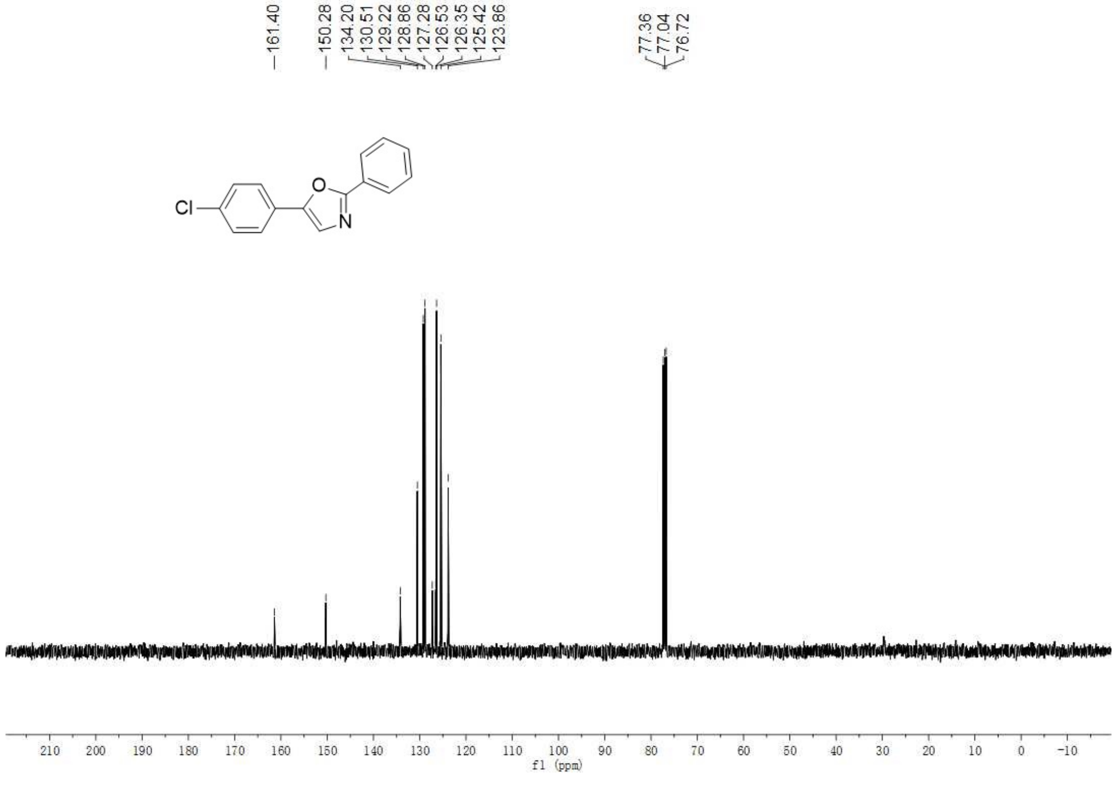
161.40
150.28
134.20
130.51
129.22
128.86
127.28
126.53
126.35
125.42
123.86
77.36
77.04
76.72
Cl
O
N
f1 (ppm)

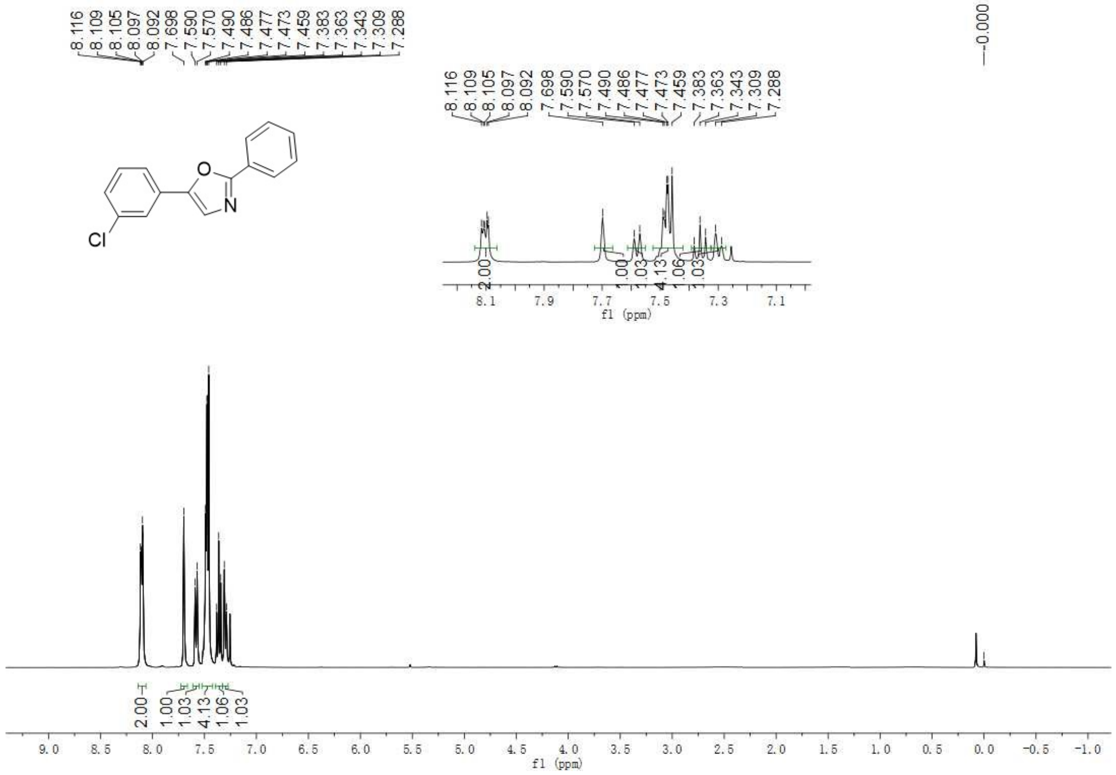
8.116
8.109
8.105
8.097
8.092
7.698
7.590
7.570
7.490
7.486
7.477
7.473
7.459
7.383
7.363
7.343
7.309
7.288
0.000
Cl
O
N
2.00
1.00
1.03
4.13
1.06
1.03
f1 (ppm)

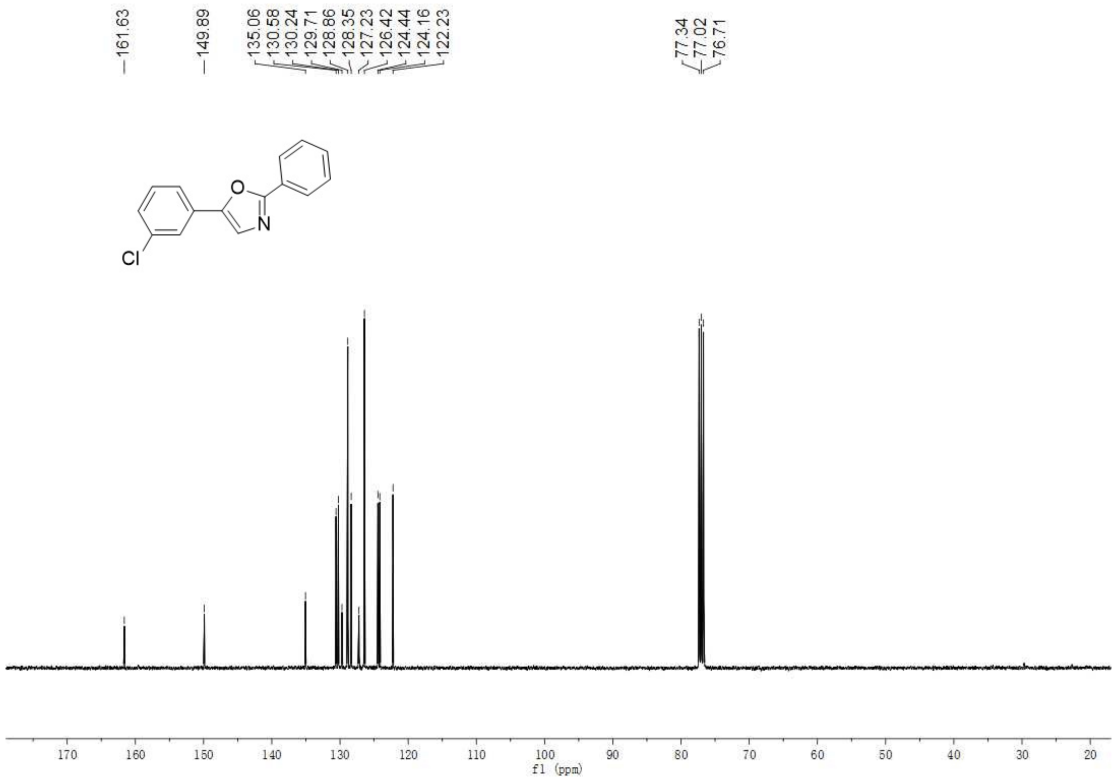
161.63
149.89
135.06
130.58
130.24
129.71
128.86
128.35
127.23
126.42
124.44
124.16
122.23
77.34
77.02
76.71
Cl
O
N
f1 (ppm)

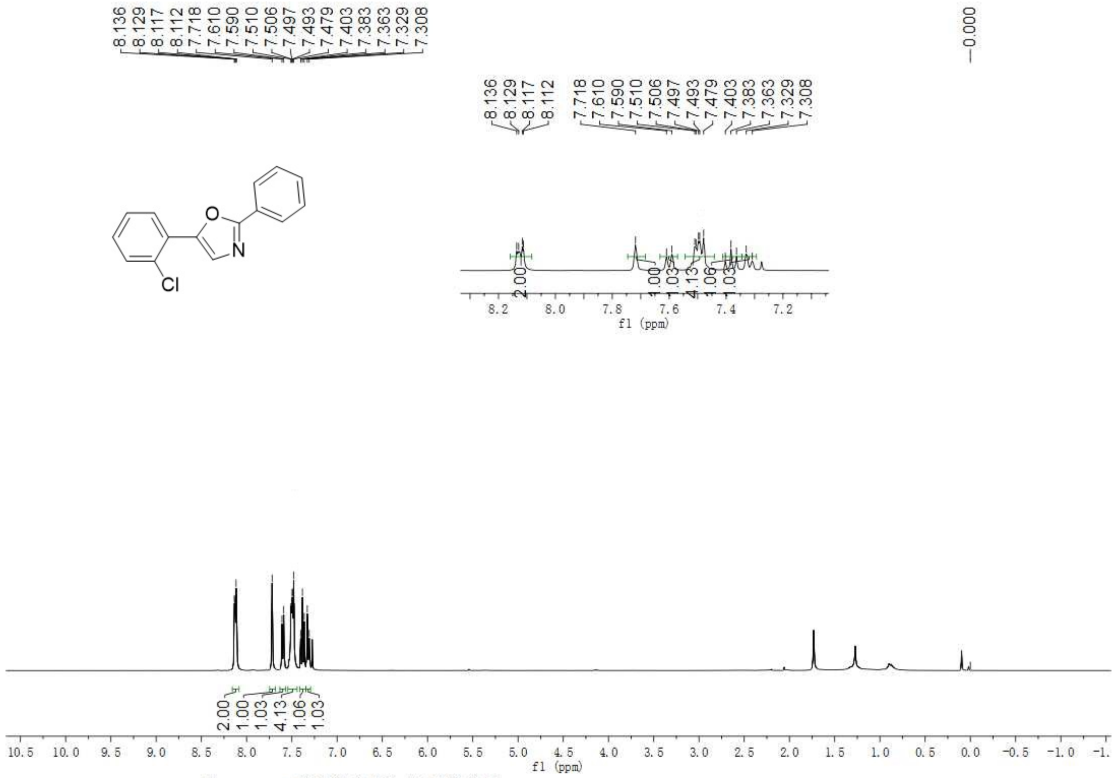
8.136
8.129
8.117
8.112
7.718
7.610
7.590
7.510
7.506
7.497
7.493
7.479
7.403
7.383
7.363
7.329
7.308
0.000
O
N
Cl
2.00
1.00
1.03
4.13
1.06
1.03
f1 (ppm)

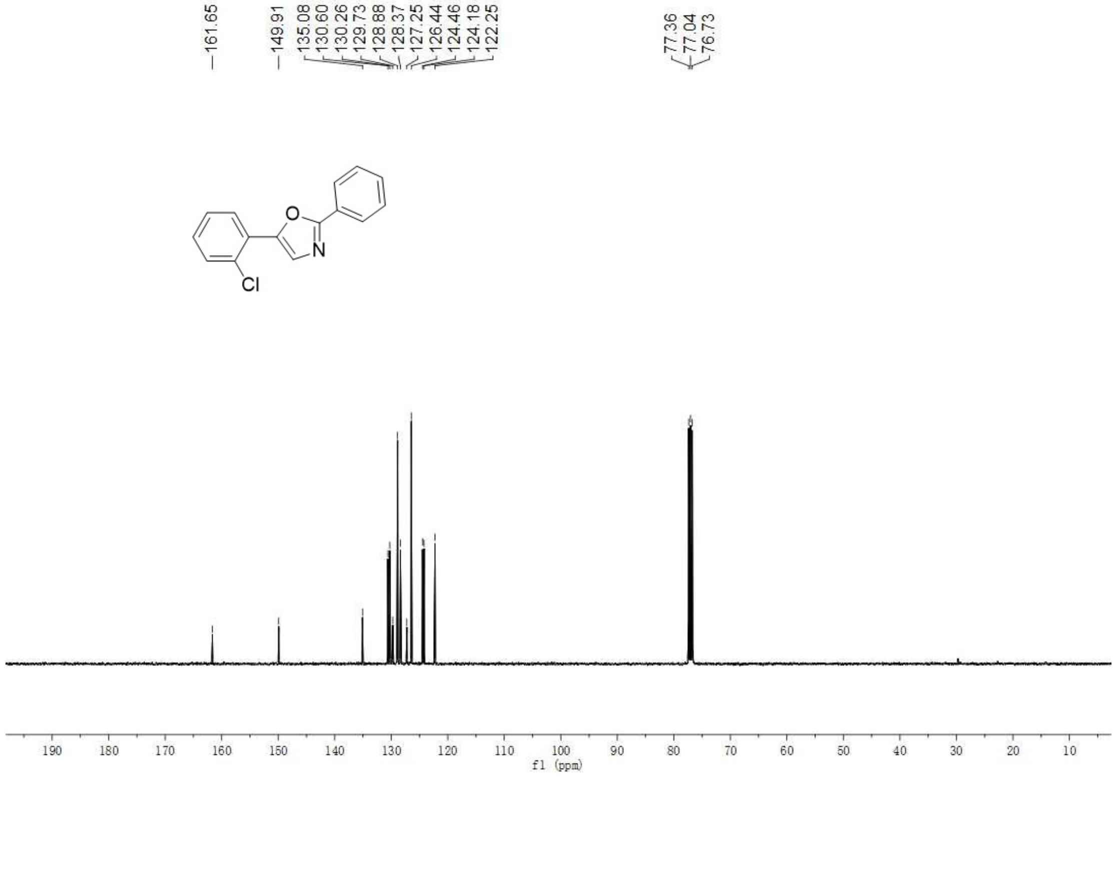
161.65
149.91
135.08
130.60
130.26
129.73
128.88
128.37
127.25
126.44
124.46
124.18
122.25
77.36
77.04
76.73
O
N
Cl
f1 (ppm)

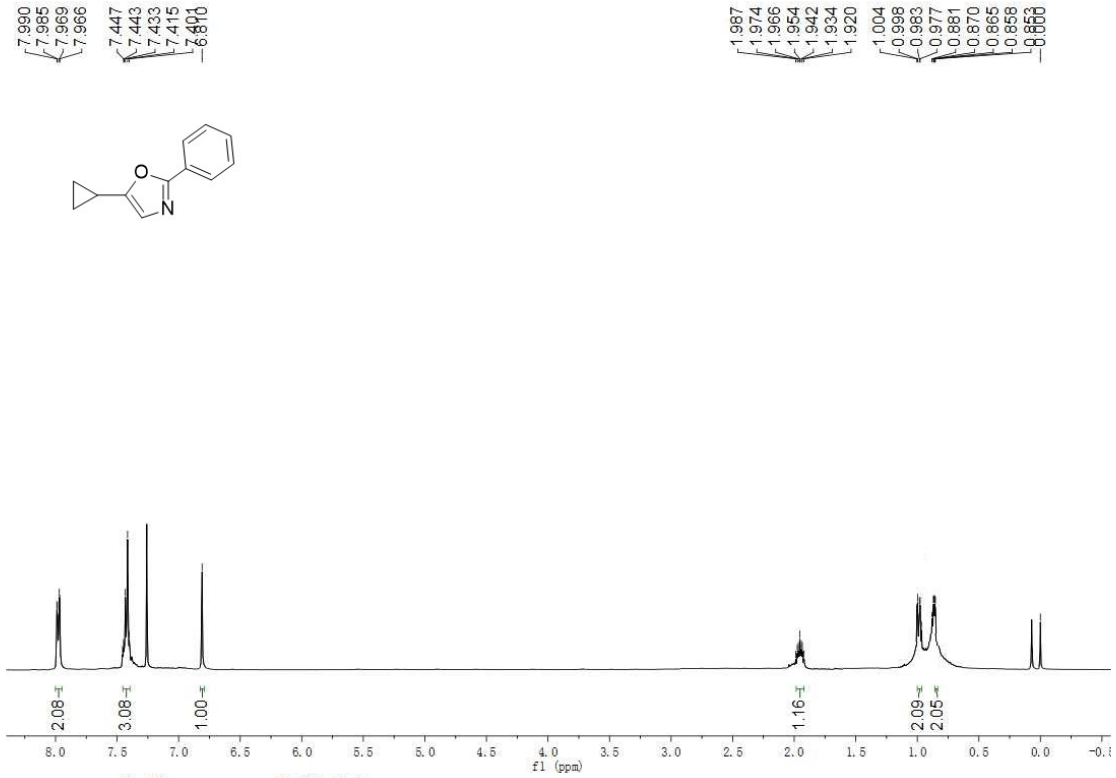
7.990
7.985
7.969
7.966
7.447
7.443
7.433
7.415
1.987
1.974
1.966
1.954
1.942
1.934
1.920
1.004
0.998
0.983
0.977
0.881
0.870
0.865
0.858
O
N
2.08
3.08
1.00
1.16
2.09
2.05
f1 (ppm)

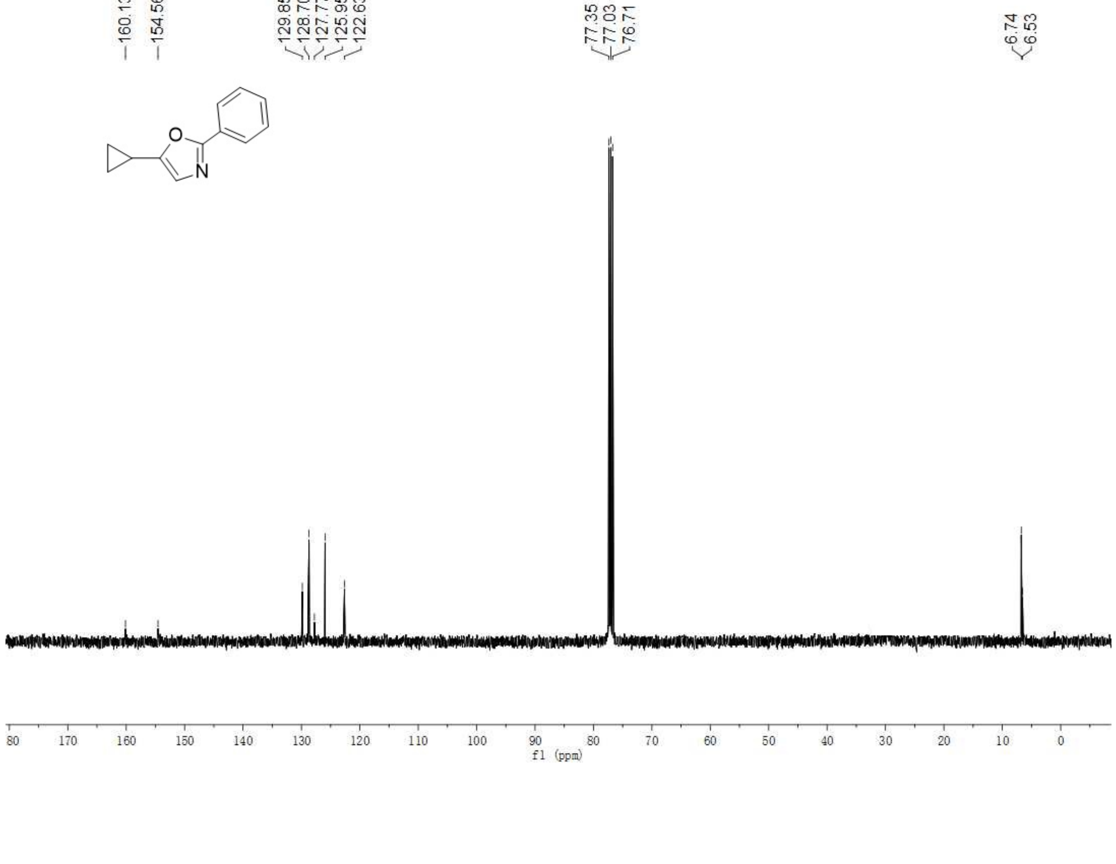
160.13
154.56
129.85
128.70
127.77
125.95
122.63
77.35
77.03
76.71
6.74
6.53
O
N
f1 (ppm)
